# NORD: NO Relaxation Delay NMR Spectroscopy

**DOI:** 10.1002/anie.202102487

**Published:** 2021-05-07

**Authors:** Tamás Milán Nagy, Katalin E. Kövér, Ole W. Sørensen

**Affiliations:** ^1^ Department of Inorganic and Analytical Chemistry University of Debrecen Egyetem tér 1 4032 Debrecen Hungary; ^2^ MTA-DE Molecular Recognition and Interaction Research Group University of Debrecen Hungary; ^3^ Copenhagen Denmark

**Keywords:** H2OBC/2BOB, heteronuclear correlation, NMR spectroscopy, NORD, SEA XLOC/HMBC

## Abstract

The novel concept of *NORD* (*NO r*elaxation *d*elay) NMR spectroscopy is introduced. The idea is to design concatenated experiments in a way that the magnetization used in the first relaxes toward equilibrium during the second and vice versa, thus saving instrument time. Applications include complete well‐resolved ^1^H‐^1^H and ^1^H‐^13^C one‐bond and long‐range correlation maps of an 80 mM solution of a trisaccharide recorded in less than two minutes and hydrocortisone with extensive spectral overlap.

## Graphical abstract

NORD (NO relaxation delay) NMR spectroscopy is introduced and combined with concatenation of experiments in a way making relaxation delays unnecessary and thus enhancing overall sensitivity.

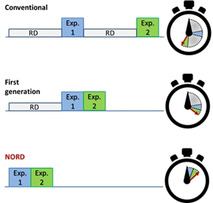

It dates back to the early days of Fourier spectroscopy in NMR when Ernst showed that parameters yielding maximum signal intensity in a single scan do not lead to maximum sensitivity when time averaging is employed.^[1]^ In fact, using an excitation flip angle of less than π/2, that is, not exciting the full available magnetization, combined with a short recovery delay typically leads to higher signal‐to‐noise ratio than when using a π/2 excitation angle and a longer recovery delay. The optimum excitation angle as a function of the T_1_ relaxation time and the delay between scans has come to be known as the Ernst angle.

This concept is in principle also applicable to complex NMR pulse sequences consisting of several pulses and delays, but it is in general not straightforward to “save” some of the initial magnetization for succeeding repetitions of the pulse sequence. An exception is where a spin echo with an excitation and a refocusing pulse is involved. If a smaller than π/2 flip angle is used for excitation the “saved” magnetization will be inverted by the refocusing pulse, so an additional π pulse is needed at the end to align the saved magnetization with the positive *z* axis and have T_1_ relaxation further increase that magnetization. Alternatively, an initial excitation angle larger than π/2 aligns the saved magnetization along the −*z* axis, so that the spin echo π pulse turns it to the +*z* axis as desired. Such ideas were used in the early days of ^13^C editing^[2]^ and more recently in connection with a couple of two‐dimensional experiments.^[3]^ However, implementation of the concept of Ernst angles in more complex NMR pulse sequences remains a challenge and possible solutions will depend on the specific pulse sequences.

This Communication demonstrates how the concept of the Ernst angle can be implemented in an efficient pulse sequence for heteronuclear correlation and implements that solution into a new concept dubbed *NORD*, *NO R*elaxation *D*elay. The idea is to have a pulse sequence containing for example, two modules representing individual experiments and laying out the overall pulse sequence such that the pool of magnetization used in the first module relaxes toward equilibrium during execution of the second and vice versa. In that way, there is no need for the usual recovery delay and instrument time can be saved.

Clearly, if the modules of the overall pulse sequence affect different spin isotopes, the solution is trivial, so the interesting case is where the modules involve the same spin isotopes, and also in a way that excludes discrimination of spins based on selective pulses.

The current work builds on recent papers on concatenated pulse sequences consisting of modules arranged so that they share a common and necessary recovery delay.^[4]^ It will be shown how to integrate NORD in all modules of such experiments. What is needed for that is suitable replacement of the π/2 excitation pulse in each module (vide infra). The modules we concatenate include HMBC^[5]^ for long‐range correlation, SEA XLOC^[6]^ yielding a spectrum similar to HMBC but with two‐ and three‐bond distinction, and 2BOB/H2OBC^[7]^ delivering an H2BC^[8]^ and a one‐bond correlation spectrum similar to HSQC.^[9]^

Two different pools of magnetization are involved in the experiments, namely ^1^H magnetization not attached (the *I* pool) or attached (the *IS* pool) to ^13^C, respectively, and the BANGO pulse sequence element^[10]^ effectively discriminates between them. Specifically, the subclass of BANGO elements of

${\left(\frac{{\pi -\beta }^{I}}{2}\right)}_{x}^{I}-\frac{1}{2J_{IS}}-{\left(\pi \right)}_{x}^{I,S}-\frac{1}{2J_{IS}}-{\left(\frac{{\pi -\beta }^{I}}{2}\right)}_{x}^{I}$

excites the *I* pool by a flip angle $\beta^{I}$ and inverts the *IS* pool by a π rotation. (Superscript on the pulse angle parentheses indicates *I* or *S* spin rf channel and subscript indicates phase.) The ^1^H π pulse later in the HMBC or SEA XLOC modules realigns the *IS* pool with the +*z* axis, and when $\beta^{I}$ is set in the range $\frac{\pi }{2}<\beta^{I}<\pi$ that is also the case for the “saved” *I* pool magnetization.

This is all pretty straightforward but the challenge arises with the succeeding 2BOB or H2OBC module using the *IS* pool. In their original forms they saturate both the *I* and the *IS* pool and thus make a recovery delay necessary before the next scan. Hence a modification that more or less leaves the *I* pool invariant and saves some of the *IS* pool magnetization is needed for NORD.

The initial part of 2BOB/H2OBC pulse sequences contains on the ^1^H channel the element ${\left(\frac{\pi }{2}\right)}_{x}-\frac{T}{2}-\pi_{x}-\frac{T}{2}-{\left(\frac{\pi }{2}\right)}_{y}$ where T is set for homonuclear coherence transfer according to pertinent *J*_HH_ (15–25 ms). Right before the second π/2 pulse the relevant product operators are in phase magnetization, $I_{y}$ , in both pools and singly antiphase magnetization, ${-2I}_{1x}I_{2z}$ , in the *IS* pool.

Disregarding NORD, the ${\left(\pi /2\right)}_{y}$ pulse is perfect in the IS pool, because it leaves $I_{y}$ invariant to yield one‐bond correlation peaks and transforms ${-2I}_{1x}I_{2z}$ into ${2I}_{1z}I_{2x}$ representing two‐bond correlation. However, to save some magnetization for the next scan a compromise pulse phase different from y is needed to turn a fraction of $I_{y}$ into ${-I}_{z}$ . That would also work for $I_{y}$ in the *I* pool but for these more slowly relaxing spins it is highly beneficial to avoid the phase compromise.

The BIG‐BIRD pulse sequence element^[11]^ can accomplish that because it allows independent phase control in the two pools. The appropriate phase difference is conveniently introduced by replacing the π/2 excitation pulse in 2BOB/H2OBC by a BIG‐BIRD version out of the following subclass:

$${\left(\frac{\pi }{4}-\frac{v}{2}\right)}_{\left(\frac{\pi }{4}-\frac{v}{2}\right)}^{I}-\frac{1}{2J_{IS}}-{\left(\pi \right)}_{x}^{I,S}-\frac{1}{2J_{IS}}-{\left(\frac{\pi }{2}\right)}_{\left(\frac{5\pi }{4}+\frac{v}{2}\right)}^{I}$$

It acts as a ${\left(\pi /2\right)}_{y}$ and a ${\left(\pi /2\right)}_{v}$ rotation in the *I* and *IS* pools, respectively, as can be verified by a vector model analysis. The one‐ and two‐bond correlation peak intensities are proportional to $cos\left(v\right)$ whilst the saved *IS* pool magnetization is proportional to$\;sin\left(v\right)$ . The saved *I* pool magnetization is not compromised for any setting of v , because all in‐phase magnetization (now present as $I_{x}$ ) is converted to ${-I}_{z}$ by the last ${\left(\pi /2\right)}_{y}^{I}$ pulse.

Figure 1 illustrates the applicable version of BIG‐BIRD with ^1^H spectra of the methyl group in sodium acetate with about 2/3 ^13^C enrichment. The central ^1^H{^12^C} resonance is clearly unaffected by the setting of the angle v . In contrast, the doublet of methyl protons attached to ^13^C shows an increasing phase shift as v is decremented from 90^0^ to 0^0^ ending up with a π/2 phase difference between the *I* and *IS* pools of magnetizations. It is a curious fact that the flip angle and the phase of the first pulse in the applied BIG‐BIRD element are always identical.

**Figure 1:**
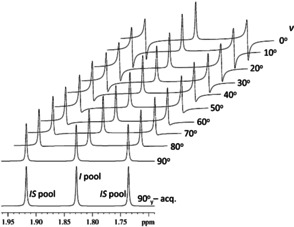
700 MHz ^1^H NMR spectra illustrating the v ‐angle dependence of the applicable BIG‐BIRD element using a 2:1 mixture of ^13^CH_3_COONa and ^12^CH_3_COONa dissolved in D_2_O and representing the *IS* (^13^CH_3_) and *I* (^12^CH_3_) pools of magnetization, respectively. The spectrum at the bottom is acquired with a single 90°_y_ excitation pulse. All the spectra were processed with identical parameters and phase correction.

The proposed NORD strategy has been implemented in the two‐module BANGO HMBC‐H2OBC experiment (see Figures S1A and S2A in ESI) and tested on a trisaccharide (**1**). The experiment performed without recovery delay between scans included only 1 scan per *t*_1_ increment. To enhance resolution the non‐uniform sampling strategy[^12^, ^13^] was utilized with 25 % NUS points in the *t*_1_ dimension. The resulting H2OBC and HMBC spectra acquired in record time of 1 min 48 s are shown in Figure 2 (for magnified view of spectra see Figure S3). The spectra yield long‐range and one‐bond information leading to unambiguous and complete ^1^H and ^13^C assignment according to the “assignment walk” shown in the H2OBC spectrum. The sequential order of the sugar residues is verified by the interglycosidic three‐bond ^1^H‐^13^C connectivities observed and indicated in the HMBC spectrum in Figure 2.

**Figure 2:**
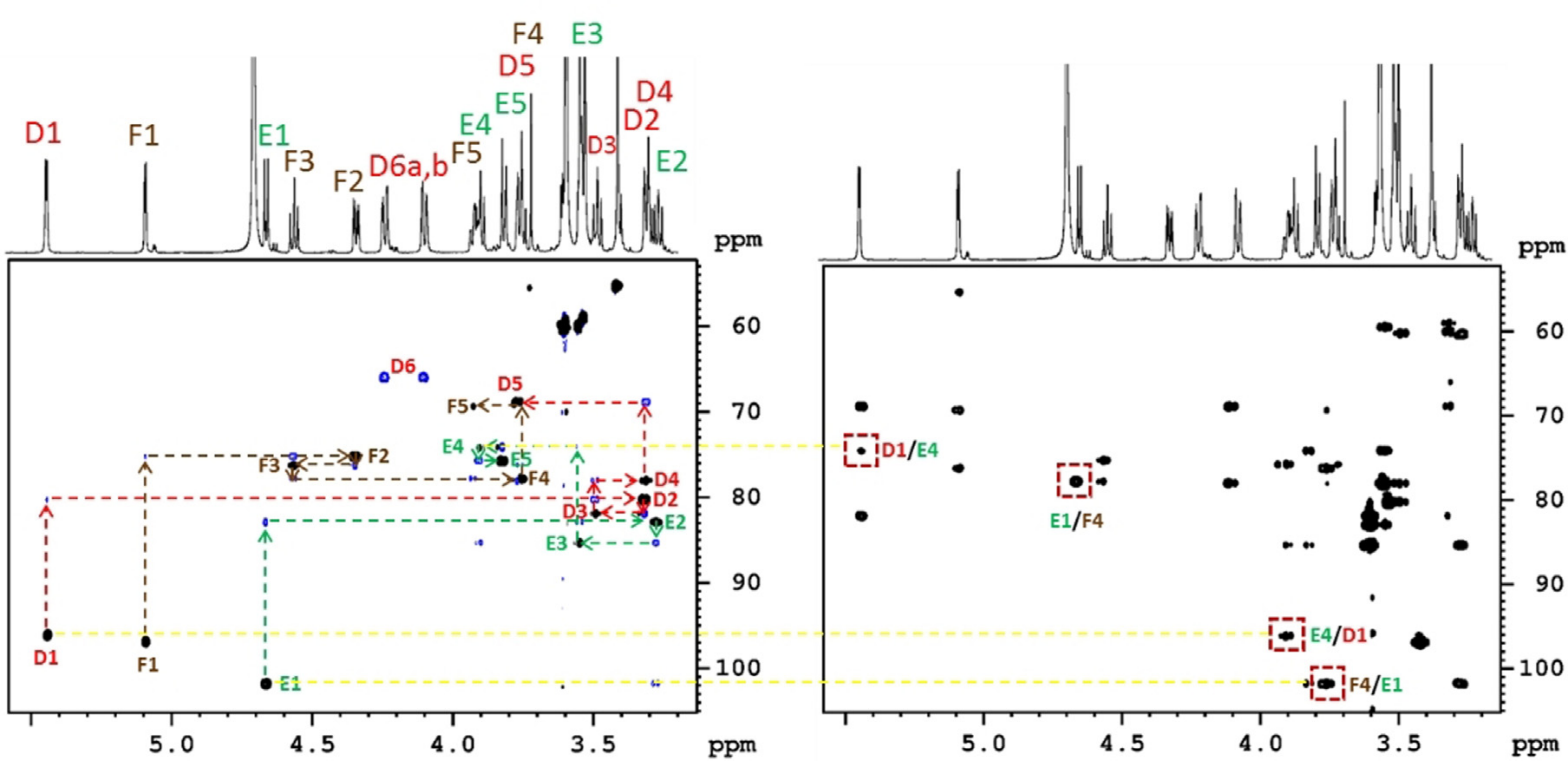
Excerpts of H2OBC (left) and HMBC spectra of a trisaccharide (**1**) (45 mg in D_2_O) recorded in 1 min 48 s on a Bruker 700 MHz Avance NEO spectrometer equipped with a TCI *z*‐gradient prodigy probe using the NORD HMBC‐H2OBC experiment with 25 % NUS. The assignment walks of D, E, and F residues are labeled by colored dotted lines in the H2OBC spectrum. Peaks framed by a red box in the HMBC spectrum verify the sequential connectivities of D–E and E–F residues. The spectra were acquired with the parameters: *Δ*=83 ms, *T*=23 ms, spectral widths of 5.1 ppm (^1^H) and 190.0 ppm (^13^C), using 64 NUS points in t_1_ with a single scan per increment and 1024 data points in t_2_. In the BANGO and BIG‐BIRD elements a CAWURST‐20(240 ppm, 1.92 ms; H2L) adiabatic ^13^C inversion pulse was used. *β* ^*I*^ was set to 120° for the BANGO pulse and the v angle of BIG‐BIRD to 20°. Before standard processing the obtained combined data was separated into HMBC and H2OBC blocks using the Bruker au‐program *splitx*. Then the non‐uniformly sampled data were reconstructed with the compressed sensing (CS) approach implemented in TopSpin and processed as in the corresponding stand‐alone experiments. The pulse sequence code for Bruker spectrometers can be found in the Supporting Information.


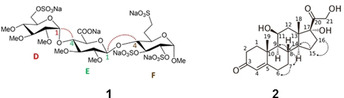


Compared to separate HMBC and H2OBC experiments the NORD approach applied to **1** showed 6–31 % sensitivity enhancements in the HMBC part. In the H2OBC part it was 22–165 % reflecting more efficient recovery of magnetization in the *IS* pool in the concatenated experiment (see Figure S4 in ESI). A shorter constant‐time delay in the H2OBC module will raise the HMBC enhancements.

Furthermore, the sensitivity enhancements achieved by the NORD approach vary with T_1_ [1] across the spectra and it is an option to attempt evening out such variations by appending an isotropic mixing[^14^, ^15^] module to NORD experiments. However, it must be kept in mind that thereby time is added, and thus the average sensitivity is reduced when there is no dominant passive magnetization reservoir to tap from.

Spectra from application of the same NORD experiment to a pentasaccharide (24 mg in 550 μL D_2_O) requiring less than 6 minutes measurement time are included in the ESI (Figure S5) and complete ^1^H and ^13^C assignments are obtained.

Key to the above NORD experiment is the BIG‐BIRD modification of H2OBC/2BOB and that can also be applied to three‐module concatenated experiments like Double BANGO SEA XLOC‐HMBC‐H2OBC/2BOB or SEA XLOC(ZQ)‐SEA XLOC(2Q)‐H2OBC/2BOB. An outline of these NORD experiments can be found in Figures S1B and S2B of the ESI.

NORD SEA XLOC‐HMBC‐2BOB was tested on hydrocortisone (**2**) (45.7 mg in 550 μL [D_6_]DMSO) exhibiting several overlapping resonances in both the proton and the carbon spectrum.

These overlaps are largely resolved by 2BOB editing into four subspectra, that is, separate one‐ and two‐bond spectra each edited according the number of attached protons being odd or even. These spectra of hydrocortisone (1B CH, 2B CH, 1B CH_2_ and 2B CH_2_) are color coded and overlaid in Figure 3 demonstrating unambiguous assignment of all ^1^H and protonated ^13^C, including the completely overlapped ones, as for example, C14‐(H14/H15a), C8‐(H8/H7a), C7‐(H7a/H8), and C15‐(H15a/H14) framed in boxes (for magnified view of spectra see Figure S6 in ESI). The “assignment walks” are indicated by arrows in the spectra. Moreover, the assignment of all quaternary carbons including two‐ and three‐bond distinction is achieved by analysis of HMBC and SEA XLOC spectra obtained in the same concatenated NORD experiment, which thereby completes the assignment of all proton and carbon resonances in hydrocortisone. These HMBC and SEA XLOC spectra with two‐ and three‐bond distinction are included in ESI (Figure S7 and Table S1).

**Figure 3:**
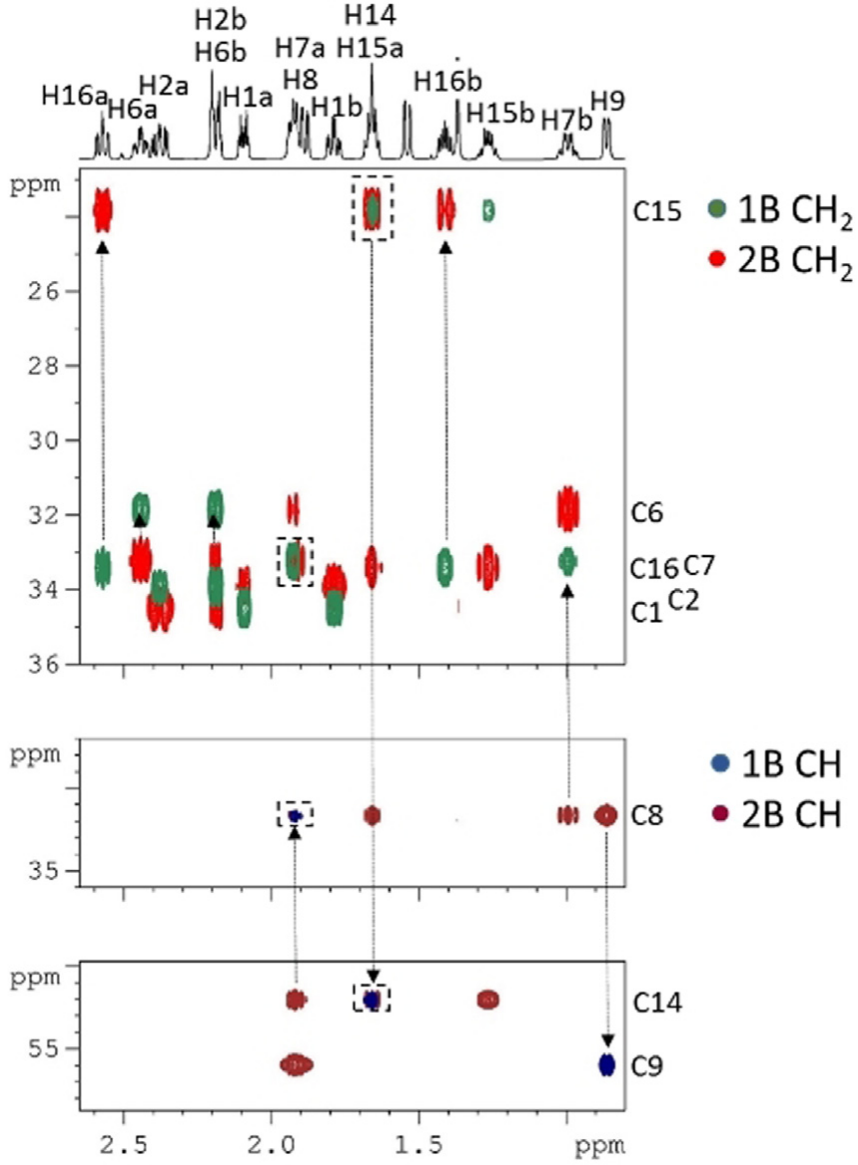
Overlay of excerpts of edited 2BOB spectra of hydrocortisone (45.7 mg in 550 μL [D_6_]DMSO) acquired using the NORD SEA XLOC‐HMBC‐2BOB experiment (outlined in Figure S1) on a Bruker Avance NEO 700 MHz spectrometer equipped with a TCI *z*‐gradient prodigy probe. One‐bond (1B) correlations of CH2 and CH carbons are plotted in green and blue, respectively, whilst the corresponding two‐bond (2B) correlations are plotted in red and brown, respectively. The experiment was performed with the following parameters: *Δ*_SEA XLOC/HMBC_=83 ms, *T*_H2OBC_=23 ms, spectral widths of 6.5 ppm (^1^H) and 190.0 ppm (^13^C), 512 points in t_1_ with a single scan per increment in the SEA XLOC and HMBC modules and 128 points with 4 scans in the 2BOB module due to the four‐step editing cycle. For all three modules 2048 data points were acquired in t_2_ amounting to 42 minutes measurement time. In the BANGO and BIG‐BIRD elements a CAWURST‐20 (240 ppm, 1.92 ms; H2L) adiabatic ^13^C inversion pulse was used. *β* ^*I*^ was set to 110° for the first and to 120° for the second BANGO, respectively, and the v angle of BIG‐BIRD to 20°. Before standard processing the obtained combined data set is separated into three blocks, corresponding to SEA XLOC, HMBC and 2BOB data, using the Bruker au‐program *splitx*. The data block of SEA XLOC is then divided further with the au‐program *split* to separate ZQ and 2Q data. The 2BOB block is divided into four data sets with the same au‐program *split* for subsequent linear combinations. Excerpts from the SEA XLOC and HMBC spectra can be found in the Supporting Information (Figure S7).

In conclusion, NORD (NO Relaxation Delay) NMR spectroscopy has been introduced in combination with concatenation of two or three experiments delivering complete heteronuclear correlation maps within minutes for small molecules. Future perspectives include concatenation of other and possibly more experiments allowing longer time for spin system recovery. That can well require development of other building blocks than BANGO and BIG‐BIRD used in this paper. Finally, it remains to be seen to what extent current and future complex NMR experiments in general can be designed or modified according to the NORD principle to exploit the concept of the Ernst angle. Where it is possible, there is potential for sensitivity gain and reduction in instrument time.

## Conflict of interest

The authors declare no conflict of interest.

## Supporting information

As a service to our authors and readers, this journal provides supporting information supplied by the authors. Such materials are peer reviewed and may be re‐organized for online delivery, but are not copy‐edited or typeset. Technical support issues arising from supporting information (other than missing files) should be addressed to the authors.

Supplementary
