# Supplementary material for: NORD: NO Relaxation Delay NMR Spectroscopy
Source: Angew Chem Int Ed Engl. 2021 May 7;60(24):13587–90. doi: 10.1002/anie.202102487 (PMC8252012; doi:10.1002/anie.202102487)
Supplement: Supplementary file 1 — Supplementary [file ANIE-60-13587-s001.pdf]

Angewandte Chemie
Eine Zeitschrift der Gesellschaft Deutscher Chemiker

Supporting Information

NORD: NO Relaxation Delay NMR Spectroscopy

Tamás Milán Nagy, Katalin E. Kövér, and Ole W. Sørensen**

anie_202102487_sm_miscellaneous_information.pdf

Table of Contents

1. NMR acronyms

2. Figure S1. Schemes for two- and three-module NORD experiments

3. Figure S2. Pulse sequences of two- and three-module NORD experiments

4. Figure S3. Magnified view of the spectra in Fig. 2

5. Figure S4. Sensitivity comparison between two-module NORD HMBC-H2OBC without relaxation delay and two separate experiments with relaxation delay, HMBC and H2OBC

6. Figure S5. Excerpts of 700 MHz H2OBC and HMBC spectra of a pentasaccharide recorded in the NORD HMBC–H2OBC two-module experiment given in Fig. S1 (A)

7. Figure S6. Magnified view of the spectra in Fig. 3

8. Figure S7. 700 MHz SEA XLOC(ZQ) and HMBC spectra of hydrocortisone recorded in the NORD SEA XLOC–HMBC–2BOB three-module experiment given in Fig. S1 (B)

9. Table S1. Complete proton (^{1}H) and carbon (^{13}C) resonance assignment of hydrocortisone in DMSO-d_6

10. Pulse sequence code of NORD HMBC–H2OBC experiment for Bruker spectrometers

11. Pulse sequence code of NORD SEA XLOC–HMBC–2BOB experiment for Bruker spectrometers

12. Pulse sequence code of NORD SEA XLOC(ZQ)–SEA XLOC(2Q)–2BOB experiment for Bruker spectrometers

13. Brainstorming on the concept of Ernst angle in complex NMR experiments

NMR acronyms

2BOB	*2-B*ond and *O*ne-*B*ond correlations
2Q	*2-Q*uantum
BANGO	small angle or β-TANGO
BIRD	*BI*linear *R*otation *D*ecoupling
BIG-BIRD	*B*iselective *I*ndependent *G*yrations BIRD
H2BC	*H*eteronuclear *2-B*ond *C*orrelation
H2OBC	*H*eteronuclear *2-B*ond and *O*ne-*B*ond *C*orrelations
HMBC	*H*eteronuclear *M*ultiple *B*ond *C*orrelation
NORD	*NO R*elaxation *D*elay
NUS	*N*on-*U*niform *S*ampling
SEA XLOC	*S*eperate *E*cho and *A*ntiecho *X* nucleus for *LO*ng-range *C*ouplings
TANGO	*T*esting for *A*djacent *N*uclei with a *G*yration *O*perator
ZQ	*Z*ero *Q*uantum

(**A**)

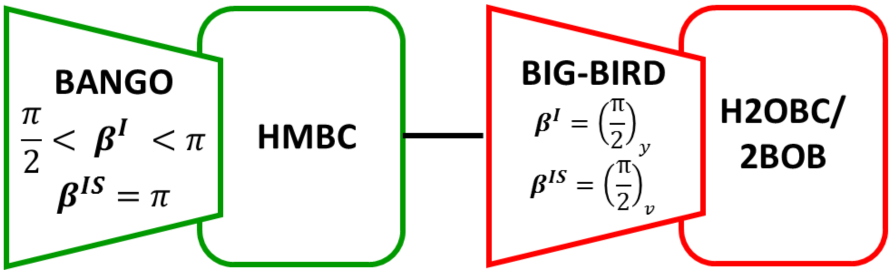

(**B**)

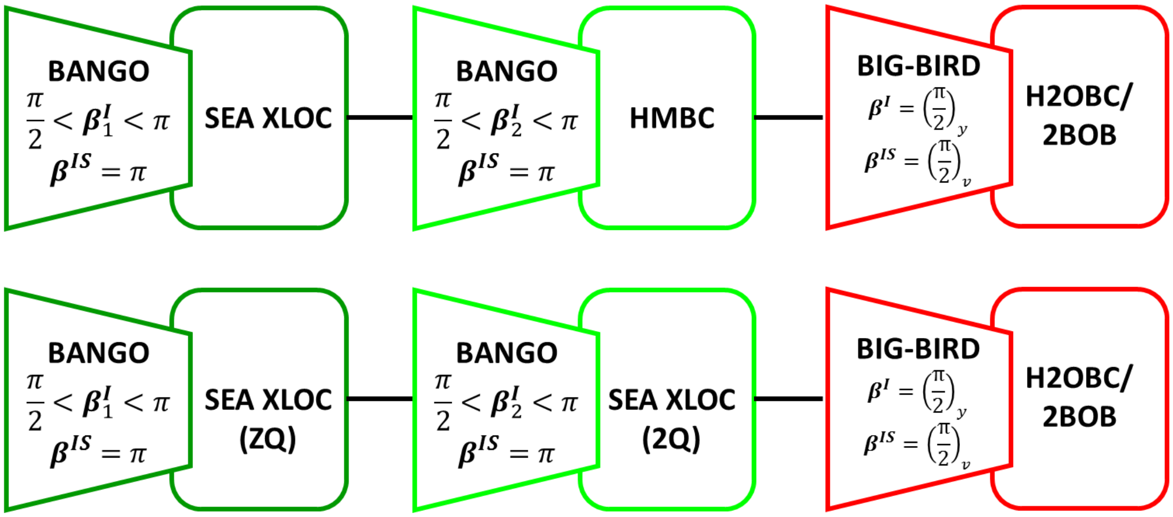

Figure S1. Schemes for two- and three-module NORD experiments (**A**) HMBC–H2OBC/2BOB utilizing a BANGO element with β^{I} of about 120° preceding the HMBC module for excitation of ^{1}H magnetization not attached to ^{13}C and a BIG-BIRD rotation with ν of about 20° preceding the H2OBC/2BOB module. (**B**) SEA XLOC–HMBC–H2OBC/2BOB (top) and SEA XLOC(ZQ)–SEA XLOC(2Q)–H2OBC/2BOB (bottom) utilizing double BANGO excitation for the first two modules sharing the pool of ^{1}H magnetization not attached to ^{13}C and BIG-BIRD rotation preceding the H2OBC/2BOB module. Prior to standard processing the obtained combined data set is separated into two (or three) blocks corresponding to the data of the two (or three) modules using the Bruker au-program *splitx*. The SEA XLOC data in (B) are further split into separate ZQ and 2Q data using the au-program *split*. The pulse sequence codes for Bruker spectrometers can be found at the end of this ESI.

(A)

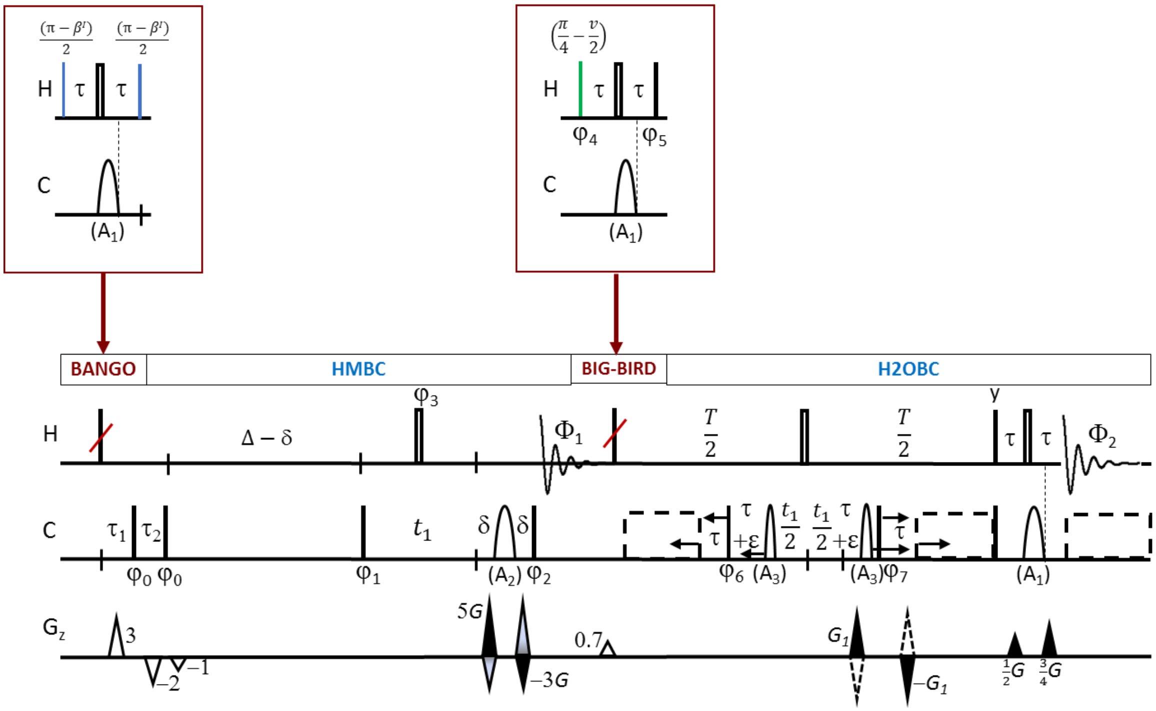

(B)

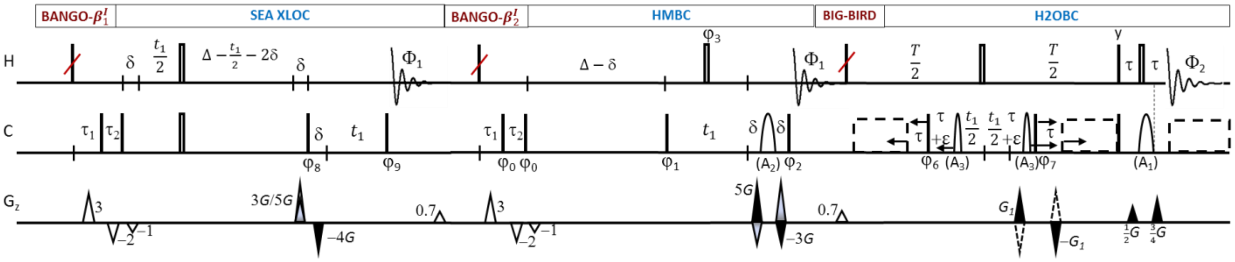

Figure S2. Pulse sequences of two- and three-module NORD experiments. NORD HMBC–H2OBC (**A**) and NORD SEA XLOC–HMBC–H2OBC (**B**). The $\pi/2$ pulses with the red lines represent the excitation pulse of the standard experiments to be replaced by the element BANGO or BIG-BIRD as indicated above them. Filled and open bars refer to $\pi/2$ and π pulses, respectively. Flip angles in the BANGO and BIG-BIRD sequences are shown above the pulses. A_1 and A_3 are *J*-compensated adiabatic inversion and refocusing pulses, CAWURST-20 (1.92 ms, 240 ppm, low-to-high frequency sweep) and CAWURST-20 (0.97 ms, 240 ppm, high-to-low frequency sweep), respectively, A_2 is composite 20% smoothed CHIRP (2 ms, 80 kHz sweep; Crp80comp.4). The BANGO and BIG-BIRD delay $\tau = 0.5(^1J)^{-1}$ is set according to 113 Hz. A 2nd order low-pass *J* filter is employed with $\tau_1 = 0.5[^1J_{min} + 0.146(^1J_{max} - {}^1J_{min})]^{-1}$ and $\tau_2 = 0.5(^1J_{max} - 0.146(^1J_{max} -$

${}^1J_{min})]^{-1}$. ε = t(π^H)/2. Δ (typically 60-100 ms) and T (typically 15-25 ms) delays are for evolution under heteronuclear long-range and homonuclear proton-proton couplings, respectively. δ is a gradient delay. The amplitude of the four purging gradients (open triangles) can be set an order of magnitude lower than the amplitude of the other ones selecting coherence transfer echo or antiecho. φ_0 = x, $\varphi_1/\varphi_6/\varphi_8$ = {x, -x, -x, x}, $\varphi_2/\varphi_7/\varphi_9$ = {x, x, -x, -x}, φ_3 = x, $\varphi_4 = \left(\frac{\pi}{4} - \frac{v}{2}\right)$, $\varphi_5 = \left(\frac{5\pi}{4} + \frac{v}{2}\right)$, and receiver phases Φ_1/Φ_2 = {x,-x}. For the current applications β^I was set to 120° for the BANGO pulse and the v angle of BIG-BIRD to 20° in (**A**). For BANGO in (**B**) β_1^I was set to 110° and β_2^I to 120°.

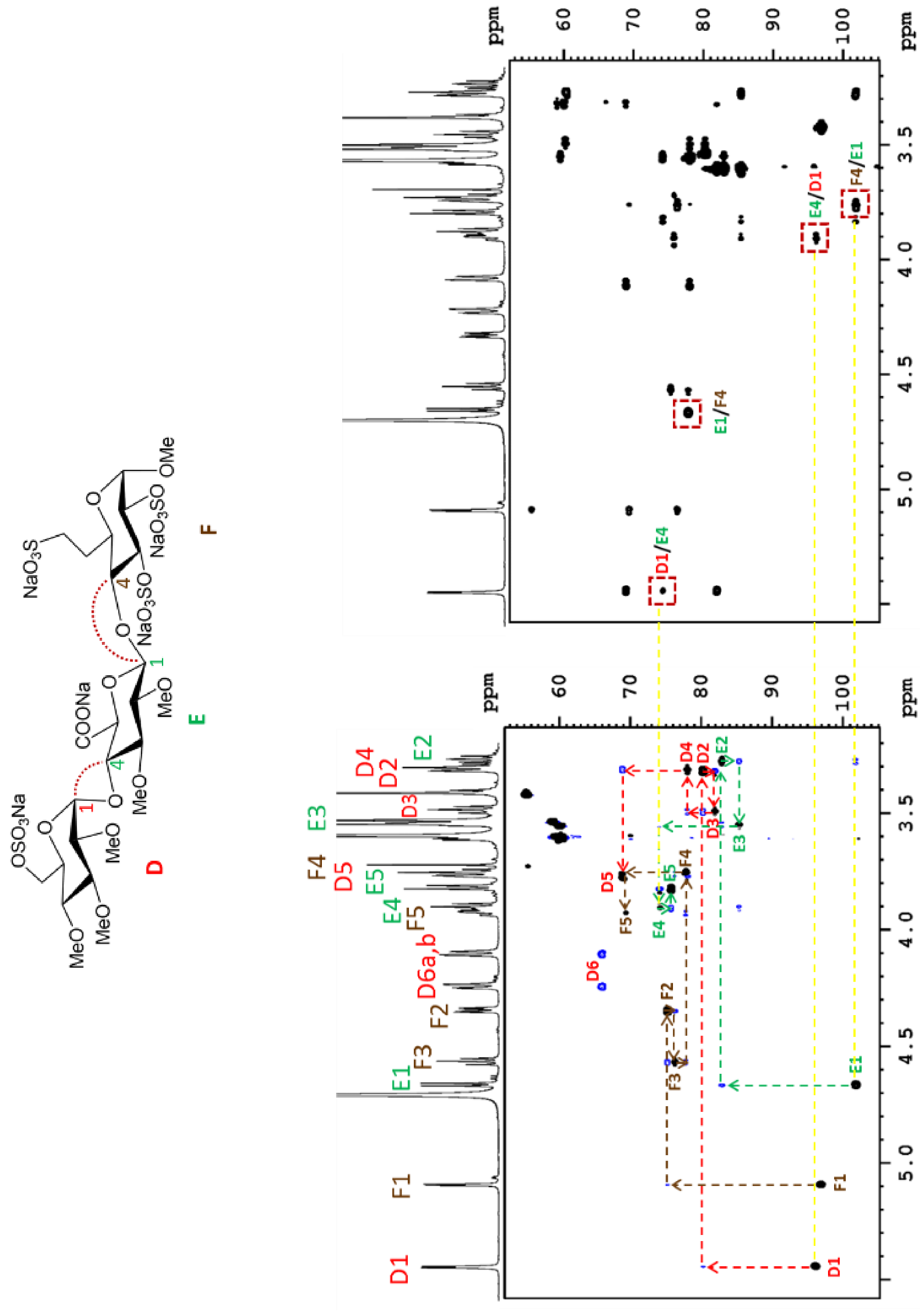

Figure S3. Magnified view of the spectra in Fig. 2.

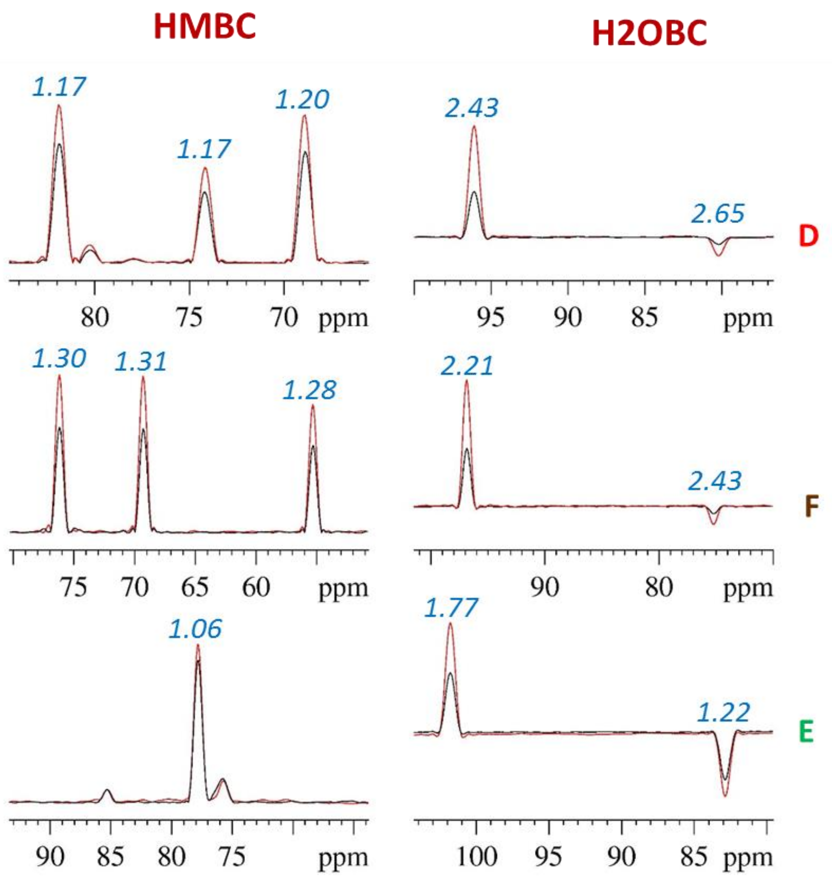

Figure S4. Sensitivity comparison between two-module NORD HMBC-H2OBC without relaxation delay (plotted in red) and two separate experiments with relaxation delay (plotted in black), HMBC and H2OBC (one- and two-bond correlation peaks positive and negative, respectively), illustrated by F_1 sections of the anomeric protons of the trisaccharide **1**. Based on sensitivity being defined as signal-to-noise ratio per unit time and bearing in mind that only relative sensitivities are of interest the following was done. With otherwise equal parameters (the same as for the spectra in Fig. 2 except for applying uniform sampling (no NUS) with 256 t_1 increments), NORD HMBC-H2OBC ran for 43 min. with 8 scans per t_1 increment and the separate experiments HMBC and H2OBC for 18 min. each with 1 scan per t_1 increment (i.e. 36 min in total for HMBC and H2OBC). Thus in order to measure the relative sensitivities directly from the corresponding peak intensities the NORD spectra are to be scaled by the factor $\sqrt{\frac{36}{43} \cdot \frac{1}{8}}$.

In practice, the factor $\sqrt{\frac{1}{8}}$ was accomplished by scaling the NORD spectra to have the noise level match the noise level in the spectra recorded separately and then the relative peak intensities were measured

from the F_1 sections shown and multiplied by the factor $\sqrt{\frac{36}{43}}$. The resulting numbers reflecting the relative sensitivities are indicated above the lines. The relaxation delay of 1.7 s represents a compromise for a relatively large spread in T_1 values for H1 and H2 protons in the figure that vary between 0.9 and 2.3 s (*D1* 1.53 s, *D2* n.a. due to overlap, *F1* 2.06 s, *F2* 2.32 s, *E1* 0.93 s, *E2* 1.86 s when attached to ^{12}C). As expected the largest enhancements are observed for the most slowly relaxing protons. Clearly, the fastest relaxing protons would benefit from a shorter relaxation delay in the separate experiments and in the NORD experiment by a larger BANGO angle β^I and a smaller BIG-BIRD angle υ. For the slowest relaxing protons it would be all opposite. Finally, the relative sensitivity enhancement in the HMBC part would be more in favor of the NORD approach for a shorter constant-time T delay in the H2OBC module, because more magnetization is saved for the succeeding scan in that way.

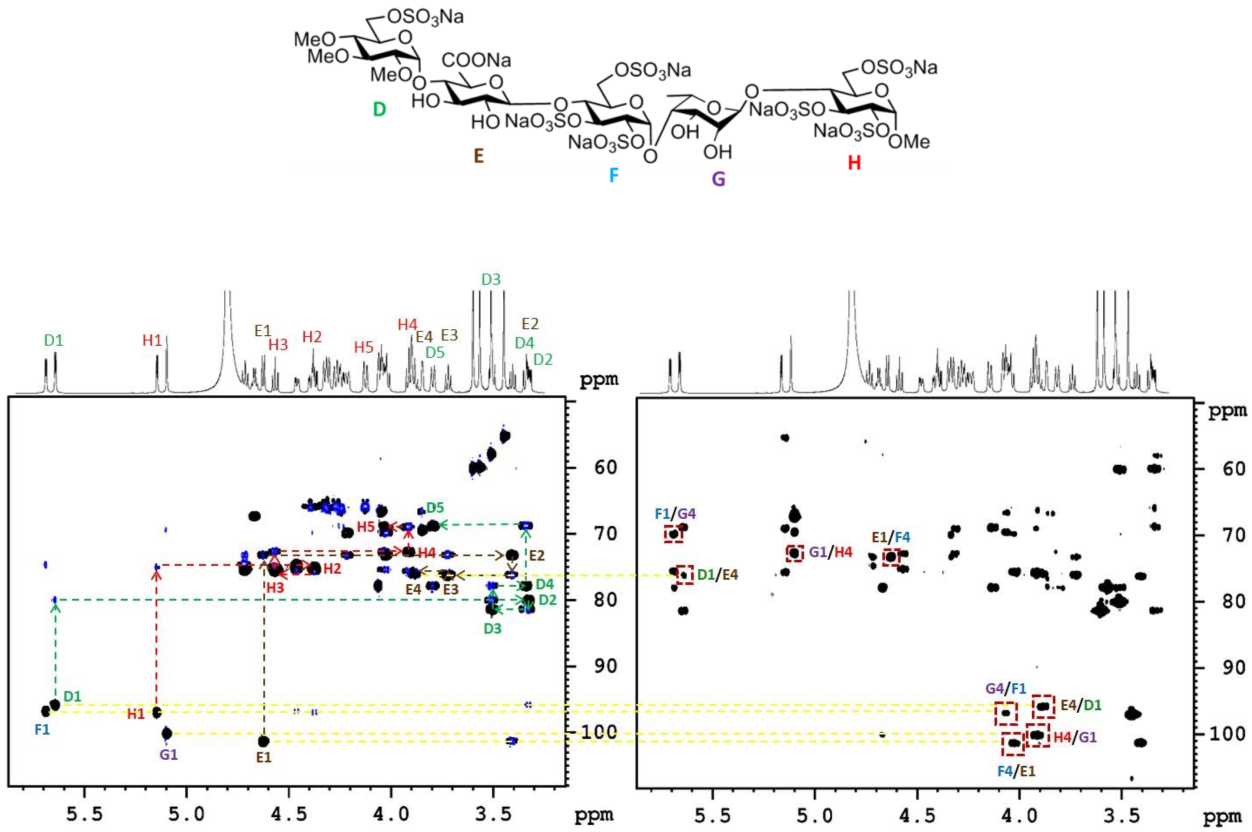

Figure S5. Excerpts of 700 MHz H2OBC (left) and HMBC (right) spectra of a pentasaccharide (24 mg in D_2O, structure shown above the spectra) recorded in less than 6 minutes using the NORD HMBC–H2OBC experiment given in Fig. S1A. The assignment walks of D, E and H residues are indicated by dotted colored lines in the H2OBC spectrum. Peaks framed in boxes in the HMBC spectrum verify the sequential connectivities of D-E, E-F, F-G and G-H residues. The spectra were acquired with the parameters: Δ = 83 ms, T = 23 ms, spectral widths of 5.1 ppm (^{1}H) and 190.0 ppm (^{13}C), using 64 NUS (25%) points in t_1 with 4 scans per increment and 1024 data points in t_2. In the BANGO and BIG BIRD excitation elements a CAWURST-20(240 ppm, 1.92 ms; H2L) adiabatic ^{13}C inversion pulse was used. β' was set to 120° in BANGO and ν in BIG-BIRD to 20°. Before standard processing the obtained combined data set is separated into two blocks, corresponding to HMBC and H2OBC data, using the Bruker au-program *splitx*. Then the non-uniformly sampled data were reconstructed with the compressed sensing (CS) approach implemented in TopSpin and processed as in their stand-alone experiments.

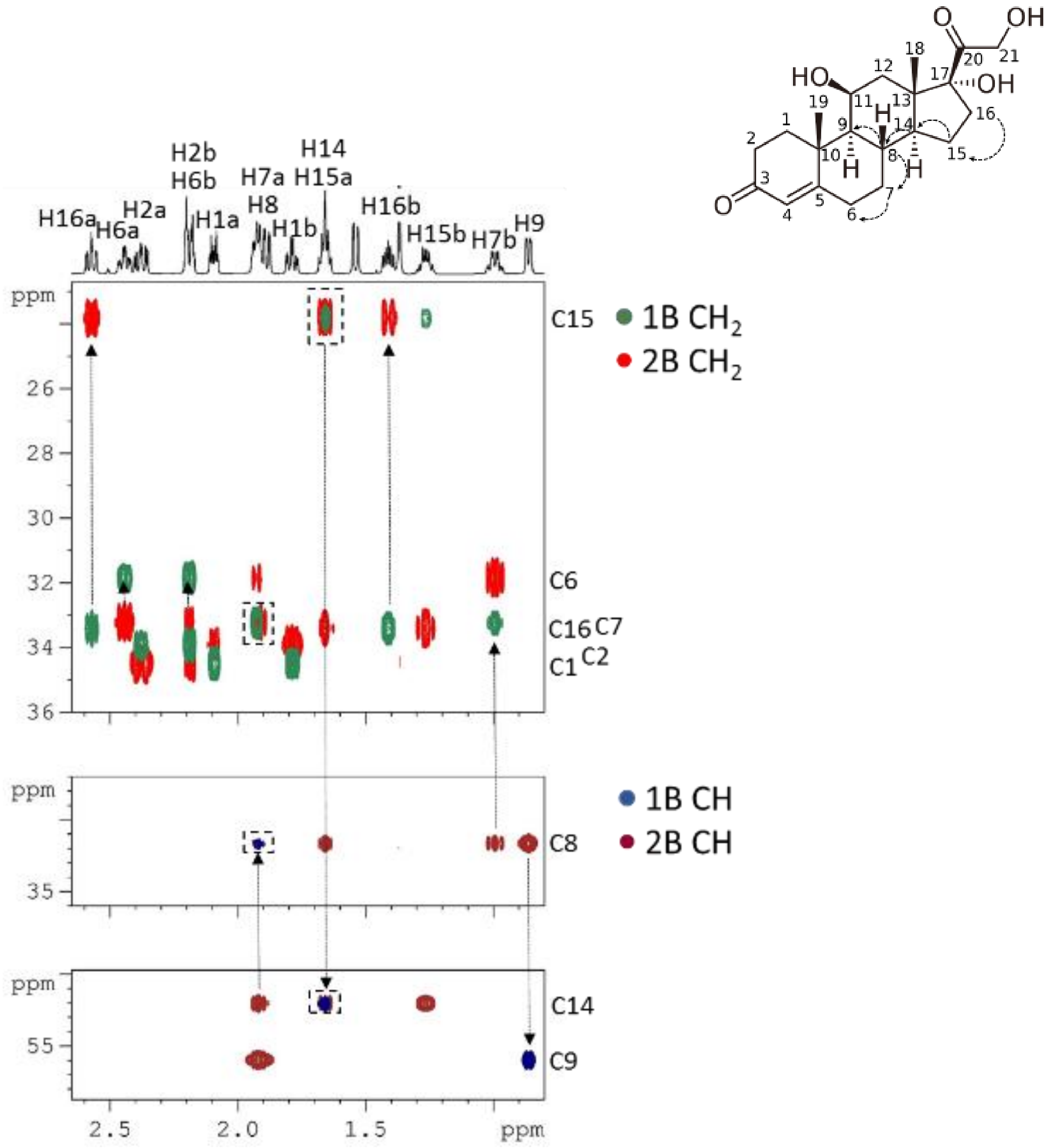

Figure S6. Magnified view of the spectra in Fig. 3.

(A)

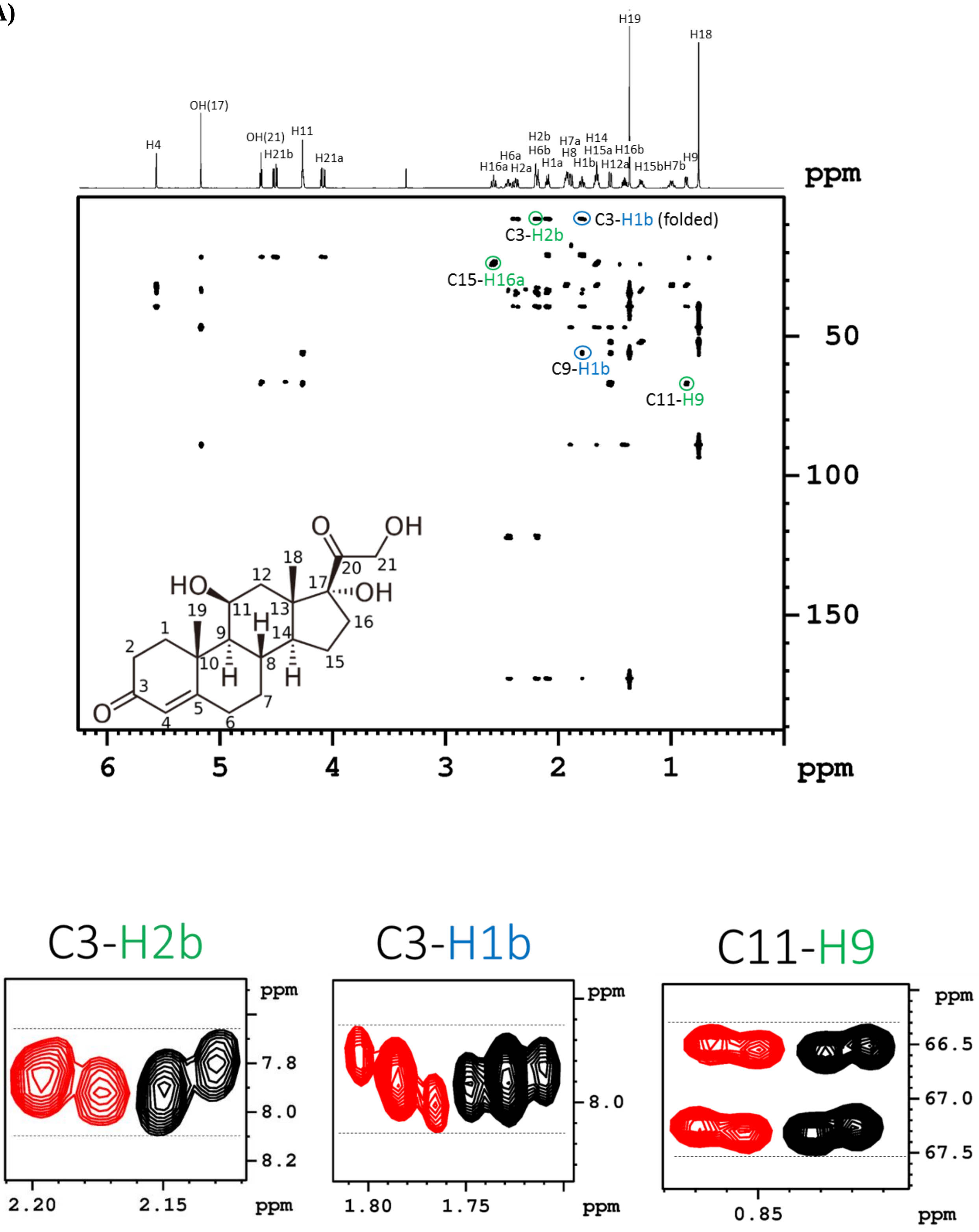

H19
H18
OH(17)
H4
OH(21)
H21b
H11
H21a
H16a
H6a
H2a
H2b
H6b
H1a
H7a
H8
H1b
H14
H15a
H12a
H16b
H15b
H7b
H9
ppm
C3-H2b
C3-H1b (folded)
C15-H16a
C9-H1b
C11-H9
50
100
150
6
5
4
3
2
1
ppm
C3-H2b
C3-H1b
C11-H9
7.8
8.0
8.2
2.20
2.15
1.80
1.75
66.5
67.0
67.5
0.85

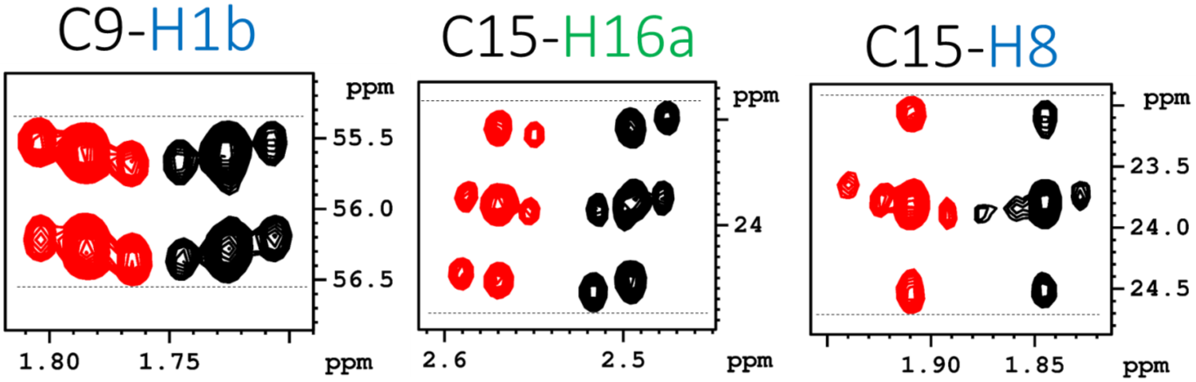

(B)

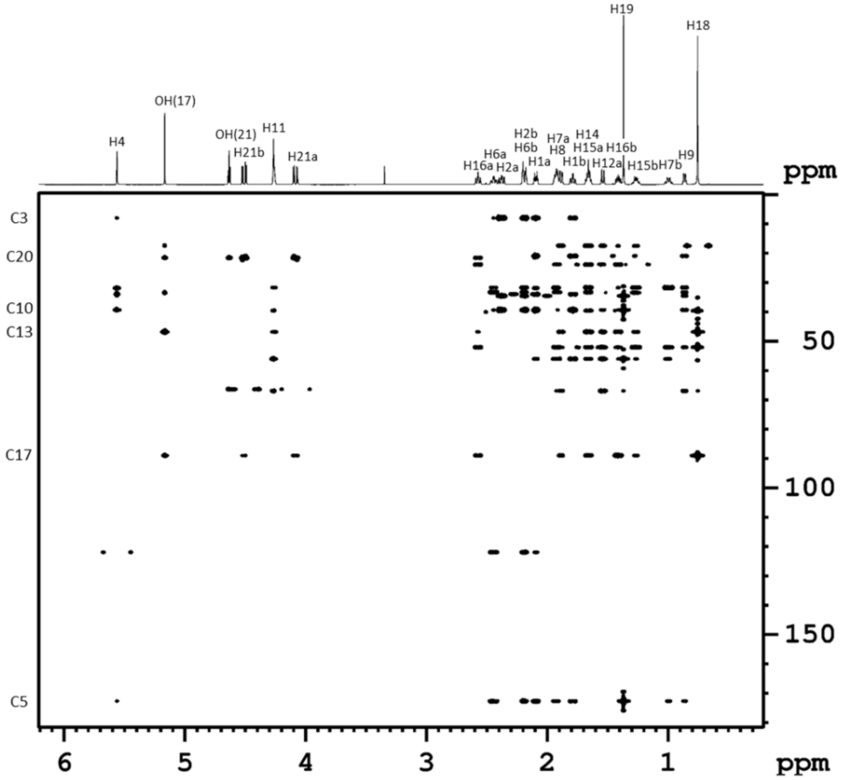

Figure S7. 700 MHz SEA XLOC(ZQ) (**A**) and HMBC (**B**) spectra of hydrocortisone recorded with the three-module experiment given in Fig. S1B using the parameters given in Fig. 4. Below the full SEA XLOC spectrum excerpts of C3 quaternary, C9, C11 and C15 protonated carbons in ZQC (red) and 2QC (black) spectra are shown (the latter displaced along the proton dimension for better visualization) and allowing to identify the number of intervening bonds as three and two on the basis of F_1 multiplet widths (*MW*) of ZQ and 2Q peaks. $MW_{ZQ} < MW_{2Q}$ indicates two-bond correlation (green), while $MW_{ZQ} > MW_{2Q}$ assigns three-bond connectivity (blue).

Table S1. Complete proton (^{1}H) and carbon (^{13}C) resonance assignment of hydrocortisone in DMSO-d_6 obatined by the combined analysis of 2BOB, HMBC and SEA XLOC spectra.

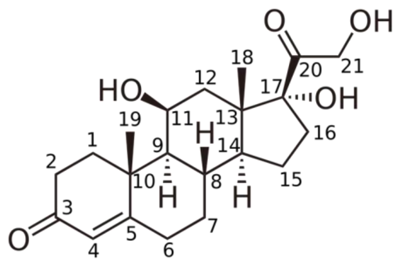

^{13}C (ppm)	^{1}H (ppm)	Assignment	Multiplicity
34.48	2.10/1.79	1	CH_2
33.89	2.38/2.19	2	CH_2
197.9	-	3	CO
121.40	5.57	4	CH
172.73	-	5	C
31.81	2.44/2.19	6	CH_2
33.22	1.92/0.99	7	CH_2
31.66	1.91	8	CH
55.88	0.86	9	CH
39.25	-	10	C
66.9	4.26	11	CH
39.1	1.89/1.54	12	CH_2
46.80	-	13	C
52.05	1.66	14	CH
23.81	1.66/1.27	15	CH_2
33.39	2.57/1.41	16	CH_2
88.90	-	17	C
17.35	0.75	18	CH_3
20.83	1.37	19	CH_3
211.46	-	20	CO
66.31	4.51/4.09	21	CH_2
-	4.26	OH(11)	-
-	4.63	OH(21)	-
	5.17	OH(17)	

Pulse sequence code of NORD HMBC–H2OBC experiment for Bruker spectrometers (*Avance III and NEO systems*)

```
;nord_hmbc_h2obc_ek_ows
;avance-version – tested on NEO system, TopSpin 4.0.2

;HMBC
;2D H-1/X correlation via heteronuclear zero- (ZQ) and double-quantum (DQ/2Q) coherence
;gradient selection of Echo/Antiecho
;with second order low-pass J-filter to suppress one-bond correlations
;no decoupling during acquisition

;H2OBC
;1B/2B heteronuclear correlation via heteronuclear zero- and double-quantum
;coherence in 13C multiplicity and 1B/2B updown mode
;gradient selection of Echo/Antiecho

;E. Kupce & O.W. Sørensen, Magn. Reson. Chem. 55, 515-518 (2017)
;N.T. Nyberg, J.Ø. Duus & O.W. Sørensen, J. Am. Chem. Soc. 127, 6154-6155 (2005)

;May 20, 2020 TMN, KEK

;This pulse sequence is part of
;Tamás Milán Nagy, Katalin E. Kövér, Ole W. Sørensen; NORD: NO Relaxation Delay NMR
;spectroscopy

;The pulse sequence has been coded for test purposes only and may contain errors.
;The functionality of the pulse sequence itself may differ depending on
;the hardware as well as the software used to execute it. Functionality
;on differing systems cannot be granted.
;Any use of this pulse sequence on a spectrometer is at your own risk.
;
;By using this pulse sequence, or any modification of it in any published material
;you agree to acknowledge the above-mentioned publication.

;NORD concatenation of TWO experiments: HMBC and H2OBC in updown mode for 13C multiplicity
;and 1B vs. 2B correlation

;BANGO and BIG-BIRD elements include J-compensated adiabatic X-inversion pulse

;set FNmode – echo-antiecho and NBL=2

;PROCESSING:
;FIRST separate the two datasets using AU splitx
;Then both datasets can be processed with xfb (set echo-antiecho, pk), then for hmbc
;use xf2m for magnitude calculation in F2


;$CLASS=HighRes
;$DIM=2D
;$TYPE=
;$SUBTYPE=
;$COMMENT=

#include <Avance.incl>
#include <Grad.incl>
#include <Delayek.incl>


;use: wvm –a in TopSpin command line to setup adiabatic pulse parameters

"cnst2=113"
```

```
"cnst30=sfo1/(sfo2*4)"
"cnst31=(1-sfo2/sfo1)/(1+sfo2/sfo1)"

define list<gradient> EA1 = {cnst30 -cnst30}    ; H2OBC, phase sensitive

define list<gradient> EA3 = { 1.000 -cnst31}    ; HMBC phase sensitive, echo-anti echo
define list<gradient> EA4 = { -cnst31 1.000}

"p2=p1*2"
"p4=p3*2"

"d0=3u"

"TAU=1s/(cnst6 + cnst7)"     ;H2OBC

"d2=1s/(cnst2*2)"            ;for J-compensated adiabatic pulse - set CNST2 = 113

"TAU9 = d2 - p29"

"TAU1=d21/2-TAU-4u-d2-d0-p2/2"   ;d21: constant time for evolution of J(HH) (16-23 ms)
"d22=TAU1"

"TAU8=d2-p16-p29-8u"
"TAU4=TAU-p16"
"d29=d21/2-d2-TAU-8u-d0-p2/2"  ;H2OBC

"in0=inf1/2"
"in22=in0"     ;H2OBC
"in29=in0"     ;H2OBC

"d6=1s/(cnst14*2)"     ;cnst14 – heteronuclear long-range coupling constant

"l3 = td1/4"    ;NBL = 2, data from two experiments are stored in one file


"DELTA1=1s/(2 * (cnst6 + 0.07 * (cnst7-cnst6) ) )"    ;H2OBC
"DELTA3=1s/(2 * (cnst7 - 0.07 * (cnst7-cnst6) ) )"
"DELTA4 = d16 + p16"
"DELTA2=(DELTA1+DELTA3)/2-DELTA4"

"DELTA6=(DELTA1-DELTA3)/2-p30/2"
"DELTA7=DELTA3-DELTA4"

"DELTA5=d2-p16-d16"
"DELTA8=d2-p16-d16-p30-8u"

"DELTA21=1s/(2 * cnst6)-p16-d16"   ;HMBC
"DELTA22=1s/(2 * cnst7)-p16-d16"
"DELTA23=d6-p16-d16-4u"
"DELTA24=p2+d10*2"


"d23=0.5/cnst2"
"d24=d23-p31"

"p13=(p1*l0/18)"      ;l0 = 7, p13 = 35 degree, corresponds to v = 20 in BIG-BIRD
                      ;set ph18 accordingly
"p11=(p1/90)*30"      ;p11 = 30 degree, corresponds to beta = 120 in BANGO


1 ze
2 d11                     ;d11=5-30m
3 d11 do:f2 st0            ;NBL = 2, d11=30 ms NORD = NO Relaxation Delay

  d11 pl2:f2

  (p3 ph1):f2
```

```
  50u UNBLKGRAD
  p16:gp12*2.77
  d16                     ;purging gradient, gpz12 = 17.13%

  (p11 ph1):f1            ;HMBC starts here with BANGO using 30 degree 1H pulse
                          ;resulting in -120 degree excitation of 1H-12C proton magn.
  d23 pl0:f2
  (p2 ph1)
  (p31:sp18 ph1):f2   ;J-compensated adiabatic pulse during BANGO - H2L sweep!
  d24 pl2:f2
  (p11 ph1):f1

  DELTA21
  p16:gp23            ;gpz23 = 15
  d16 pl2:f2
  (p3 ph23):f2
  DELTA22
  p16:gp14            ;gpz14 = -10
  d16
  (p3 ph23):f2
  4u
  p16:gp15            ;gpz15 = -5
  d16
  DELTA23
  (p3 ph24):f2
  d0
  (p2 ph22):f1
  d0
  p16:gp1*EA3          ;gpz1 = 80
  d16
  (p24:sp7 ph25):f2     ;p24 = 2msec for Crp80comp.4
  DELTA24
  p16:gp1*EA4
  d16 pl2:f2
  (p3 ph25):f2
  4u BLKGRAD

  goscnp ph30


;second experiment starts here – H2OBC

22 d11 st        ;d11=5-30m
23 d11 do:f2     ;d11 only short delay between experiments
d11 pl2:f2

(p3 ph1):f2
50u UNBLKGRAD

p16:gp12*1.77           ;purging gradient between experiments
d16                     ;gpz12 = 17.13%

20u

(p13 ph19):f1        ;p13 = (p1*l0/18) and l0 = 7, corresponds to v = 20 degree in BIG-BIRD
d23 pl0:f2
(p2 ph1)
(p31:sp18 ph1):f2    ;J-compensated adiabatic pulse during BIG-BIRD - H2L sweep!
d24 pl12:f2
(p1 ph20):f1

d22 cpd2:f2            ;with 13C-multiplicity editing
TAU do:f2
4u pl2:f2
(p3 ph3):f2

TAU9 pl0:f2         ;TAU9 = d2 - p29
```

```
(p29:sp20 ph1):f2   ;J-compensated adiabatic pulse - L2H sweep, 0.97ms!
d0
(p2 ph1)
d0
p16:gp1*EA1
TAU8
4u
(p29:sp20 ph1):f2   ;J-compensated adiabatic pulse - L2H sweep, 0.97ms!
4u pl2:f2
(p3 ph5):f2
p16:gp1*EA1*-1
TAU4 pl12:f2
d29 cpd2:f2

4u do:f2
4u pl2:f2

(p1 ph2) (p3 ph1):f2

p16:gp1*0.5
d16
DELTA5 pl0:f2
(p2 ph1)
(p30:sp19 ph1):f2   ;J-compensated adiabatic pulse during editing - H2L sweep, 1.92 ms
p16:gp1*0.75
d16
DELTA8 pl12:f2
4u cpd2:f2
4u BLKGRAD

go=2 ph31
d11 do:f2 wr #0 if #0 zd

d11*0.33 igrad EA1
d11*0.33 igrad EA3
d11*0.33 igrad EA4

lo to 2 times 2

d11*0.15 id0
d11*0.15 dd22
d11*0.15 dd29

d11*0.15 ip3*2

d11*0.15 ip24*2
d11*0.15 ip30*2
d11*0.15 ip31*2

lo to 2 times l3    ;l3 = td1/4,  NBL = 2


exit

ph1=0
ph14=0 2 2 0
ph15=0 0 2 2

ph2=1
ph3=0 0 2 2
ph5=0 2
ph6=0

ph22=0 0 0 0 2 2 2 2
ph23=0 0 2 2
ph24=0 2
ph25=0 0 0 0 0 0 0 0 2 2 2 2 2 2 2 2
```

```
ph16=(8) 1   ;BIG-BIRD phases - ph16-ph20
ph17=(8) 5

ph18=(72) 2

ph19 = ph16 - ph18
ph20 = ph17 + ph18

ph30=0 2 0 2 0 2 0 2 2 0 2 0 2 0 2 0
ph31=0 2 2 0



;pl1 : f1 channel - power level for pulse (default)
;pl2 : f2 channel - power level for pulse (default)
;pl12: f2 channel - power level for CPD/BB decoupling
;p1 : f1 channel - 90 degree high power pulse
;p2 : f1 channel - 180 degree high power pulse
;p3 : f2 channel - 90 degree high power pulse
;p16: homospoil/gradient pulse [1 msec]
;p24: f2 channel - 180 degree shaped pulse for refocussing
; = 2msec for Crp60comp.4
;d0 : incremented delay (2D) [3 usec]
;d11: delay for disk I/O [5-30 msec]
;d16: delay for homospoil/gradient recovery

;d22: decremented delay (2D)
;d29: decremented delay (2D)
;d21: constant time (evolution of J(HH)) [16 - 23 msec]

;cnst2: = 113
;cnst6: = 1J(XH)min
;cnst7: = 1J(XH)max
;cnst14: = J(XH) long range

;inf1: 1/SW(X) = 2 * DW(X)
;in0: 1/(2 * SW(X)) = DW(X)
;in22: = in0
;in29: = in0
;nd0: 2
;ns: 2 * n
;ds: >= 16
;td1: number of experiments
;FnMODE: echo-antiecho


;cpd2: decoupling according to sequence defined by cpdprg2
;pcpd2: f2 channel - 90 degree pulse for decoupling sequence

; ~~~~~~~~~~~~~~~ WaveMaker Shapes ~~~~~~~~~~~~~~~~~~~~~~~~~~~
;USE: wvm -a  command in TopSpin command line to generate CA-WURST adiabatic shape pulses

;sp18:wvm:ad180Ccawurst: cawurst-20(240 ppm, 1.92 ms; H2L)
;sp19:wvm:ad180Ccawurst: cawurst-20(240 ppm, 1.92 ms; H2L)
;sp20:wvm:ad180Ccawurstlh2: cawurst-20(240 ppm, 0.97 ms; L2H)


;for z-only gradients:
;gpz1: 80%
;gpz2: -40.1% for C-13
;gpz3:  40.1%
;gpz4: 60%
;gpz5: -40%
;gpz6: -20%
;gpz12 = 17.13% purging gradient
;gpz23 = 15%
```

```
;gpz14= -10%
;gpz15= -5%

;use gradient files:
;gpnam: SMSQ10.100
```

//

Pulse sequence code of NORD SEA XLOC–HMBC–2BOB(with 4-step phase editing) experiment for Bruker spectrometers (*Avance III and NEO systems*)

```
;nord_sea-xloc_hmbc_2bob(4)_ek_ows
;avance-version – tested on NEO system, TopSpin 4.0.2

;SEA XLOC
;2D H-1/X correlation via heteronuclear zero- (ZQ) or double-quantum (DQ/2Q) coherence
;recorded in two separate (SEA = separate echo-antiecho) experiments
;optimized for long range couplings
;with second order low-pass J-filter to suppress one-bond correlations
;no decoupling during acquisition
;using gradient pulses for coherence selection
;using adiabatic shaped pulses for inversion on f2 - channel

;T. Gyongyosi, T.M. Nagy, K.E. Kover & O.W. Sørensen, Chem. Commun. 54, 9781-9784 (2018)

;HMBC
;2D H-1/X correlation via heteronuclear zero (ZQ) or double quantum (DQ/2Q) coherence
;phase sensitive using Echo/Antiecho gradient selection
;with second order low-pass J-filter to suppress one-bond correlations
;no decoupling during acquisition

;2BOB
;1B/2B heteronuclear correlation via heteronuclear zero- and double-quantum
;coherence with 13C-multiplicity and 1B/2B up-down time-shared 4-step phase cycle and
;Echo/Antiecho gradient selection

;E. Kupce & O.W. Sørensen, Magn. Reson. Chem. 55, 515-518 (2017)
;N.T. Nyberg, J.Ø. Duus & O.W. Sørensen, J. Am. Chem. Soc. 127, 6154-6155 (2005)

;May 20, 2020 TMN, KEK

;This pulse sequence is part of
;Tamás Milán Nagy, Katalin E. Kövér, Ole W. Sørensen; NORD: NO Relaxation Delay NMR
;spectroscopy

;The pulse sequence has been coded for test purposes only and
;may contain errors.
;The functionality of the pulse sequence itself may differ depending on
;the hardware as well as the software used to execute it. Functionality
;on differing systems cannot be granted.
;Any use of this pulse sequence on a spectrometer is at your own risk.
;
;By using this pulse sequence, or any modification of it in any published material
;you agree to acknowledge the above-mentioned publication.

;NORD concatenation of THREE experiments: SEA XLOC, HMBC and 2BOB with time-shared 4-step
;phase cycle of correlations according to 13C-multiplicity and 1B vs. 2B correlation

;Double BANGO and BIG-BIRD elements include J-compensated adiabatic X-inversion pulse
```

```
;set FNmode - UNDEFINED and NBL=3


;PROCESSING:

;FIRST separate the three datasets using AU splitx
;Then separate the first (SEA XLOC) data set with AU split into 2Q and ZQ data - then
;process both with xfb (set QF, mc in F1)
;The second data set corresponds to HMBC and can be processed with xfb (set echo-
;antiecho, pk, then use xf2m for magnitude calculation in F2)
;The third data set corresponds to 2BOB with four-step phase cycle: 1B vs. 2B and also
;with carbon multiplicity distinction, before processing use AU split (row 4!)
;Then the resulting four data sets can be processed with xfb (set echo-antiecho, pk)
;NOTE: F1 resolution in HMBC and SEA XLOC is four times higher than that of 2BOB

;$CLASS=HighRes
;$DIM=2D
;$TYPE=
;$SUBTYPE=
;$COMMENT=

#include <Avance.incl>
#include <Grad.incl>
#include <Delayek.incl>


;use: wvm –a in TopSpin command line to setup adiabatic pulse parameters

"cnst2=113"

"cnst30=sfo1/(sfo2*4)"
"cnst31=(1-sfo2/sfo1)/(1+sfo2/sfo1)"

define list<gradient> EA1 = {cnst30 -cnst30}    ; 2BOB, phase sensitive
define list<gradient> EA2 = {30 50}             ; ZQ- and 2Q-selection in SEA XLOC
define list<gradient> EA3 = { 1.000 -cnst31}    ; HMBC phase sensitive, echo-anti echo
define list<gradient> EA4 = { -cnst31 1.000}
define list<gradient> EA5 = { 0.5 0.5 0.25 0.25}


"p2=p1*2"
"p4=p3*2"

"d0=3u"                        ;d0 for 2BOB
"d10=3u"                       ;d10 for HMBC and SEA XLOC

"l9=0"
"l8=0"

"TAU=1s/(cnst6 + cnst7)"     ;2BOB

"d2=1s/(cnst2*2)"            ;for J-compensated adiabatic pulse - set CNST2 = 113

"TAU9 = d2 - p29"

"TAU1=d21/2-TAU-4u-d2-d0-p2/2"   ;d21: constant time for evolution of J(HH) (16-23 ms)
"d22=TAU1"

"TAU2=d21/2-TAU-p3-d0-p2/2-4u"   ;without 13C editing
"d24=TAU2"

"DELTA=d0*2+p2"
"TAU3=d21/2-p2/2-p3-TAU-d0-p16*2-d16*2-p29*2-DELTA-16u"   ;without 13C editing
"d25=TAU3"

"TAU8=d2-p16-p29-8u"
"TAU4=TAU-p16"
```

```
"d29=d21/2-d2-TAU-8u-d0-p2/2"  ;2BOB

"in0=inf1/2"
"in10=inf1/2"
"in19=in0"     ;SEA XLOC

"in22=in0"     ;2BOB
"in29=in0"     ;2BOB
"in24=in0"    ;2BOB
"in25=in0"    ;2BOB

"d6=1s/(cnst14*2)"     ;cnst14 – heteronuclear long-range coupling constant

"l3 = td1/24"    ;NBL = 3, data from three experiments are stored in one file

"DELTA1=1s/(2 * (cnst6 + 0.07 * (cnst7-cnst6) ) )"    ;2BOB
"DELTA3=1s/(2 * (cnst7 - 0.07 * (cnst7-cnst6) ) )"
"DELTA4 = d16 + p16"
"DELTA2=(DELTA1+DELTA3)/2-DELTA4"

"DELTA5=d2-p16-d16"
"DELTA8=d2-p16-d16-p30-8u"
"DELTA7=d2-p16-d16-8u"    ;without 1B/2B editing

"DELTA9=1s/(2 * (cnst6 + 0.146 * (cnst7-cnst6)) ) -p16-d16"  ;SEA XLOC
"DELTA12=1s/(2 * (cnst7 - 0.146 * (cnst7-cnst6)) ) -p16-d16"

"DELTA21=1s/(2 * cnst6)-p16-d16"   ;HMBC
"DELTA22=1s/(2 * cnst7)-p16-d16"
"DELTA23=d6-p16-d16-4u"
"DELTA24=p2+d10*2"

"d23=1s/(cnst14*4)"
"d19=d23*2-p16*2-d16*2-d10"

"d26=0.5/cnst2"
"d27=d26-p31"

"p13=(p1*l0/18)"      ;l0 = 7, p13 = 35 degree, corresponds to v = 20 in BIG-BIRD
                      ;set ph18 accordingly
"p11 = (p1/90)*30"    ;p11 = 30 degree, corresponds to beta = 120 in second BANGO
"p12 = (p1/90)*35"    ;p12 = 35 degree, corresponds to beta = 110 in first BANGO



1 ze
2 d11 do:f2                   ;d11=5-30 ms
  6m pl12:f2
3 5m
4 2m
5 7m do:f2
6 50u pl1:f1

  d11 do:f2 st0               ;NBL = 3, d11=5-30 ms NORD = NO Relaxation Delay

  d11 pl2:f2

  (p3 ph1):f2
  50u UNBLKGRAD
  p16:gp12*2.77
  d16                         ;purging gradient, gpz12 = 17.13%

  (p12 ph1):f1                ;SEA XLOC starts here with BANGO using 35 degree 1H pulse
                              ;resulting in -110 degree excitation of 1H-12C proton magn.
  d26 pl0:f2
  (p2 ph1)
  (p31:sp18 ph1):f2           ;J-compensated adiabatic pulse during BANGO - H2L sweep!
```

```
  d27
  (p12 ph1):f1

  DELTA9
  p16:gp4
  d16 pl2:f2
  (p3 ph1):f2
  DELTA12
  p16:gp5
  d16
  (p3 ph1):f2
  p16:gp6
  d16 pl2:f2
  d10
  (center (p2 ph1) (p4 ph1):f2 )
  d19
  p16:gp11*EA2               ;gpz11 = 1 multiplied by 30 for ZQ- or 50 for 2Q-selection
  d16
 (p3 ph14):f2
  p16:gp2
  d16 BLKGRAD
  d10
  d10
 (p3 ph15):f2

  goscnp ph29

 ;second experiment starts here: HMBC phase sensitive


12  d11 do:f2 st      ;d11=5-30 ms
13  d11 pl2:f2

  (p3 ph1):f2

  50u UNBLKGRAD
  p16:gp12*2.77               ;purging gradient between experiments
  d16                         ;gpz12 = 17.13%


  (p11 ph1):f1       ;HMBC starts here with BANGO using 30 degree 1H pulse resulting in
                     ;-120 degree excitation of 1H-12C proton magn.
  d26 pl0:f2
  (p2 ph1)
  (p31:sp18 ph1):f2   ;J-compensated adiabatic pulse during BANGO - H2L sweep!
  d27 pl2:f2
  (p11 ph1):f1

  DELTA21
  p16:gp23             ;gpz23 = 15
  d16 pl2:f2
  (p3 ph23):f2
  DELTA22
  p16:gp14             ;gpz14 = -10
  d16
  (p3 ph23):f2
  4u
  p16:gp15             ;gpz15 = -5
  d16
  DELTA23
  (p3 ph24):f2
  d10
  (p2 ph22):f1
  d10
  p16:gp1*EA3          ;gpz1 = 80
  d16
  (p24:sp7 ph25):f2    ;p24 = 2msec for Crp80comp.4
```

```
  DELTA24
  p16:gp1*EA4
  d16 pl2:f2
  (p3 ph25):f2
  4u BLKGRAD

  goscnp ph30


;third experiment starts here - 2BOB with 4-step time-shared phase editing


22 d11 st        ;d11=5-30 ms
23 d11 do:f2     ;d11 only short delay between experiments
d11 pl2:f2

(p3 ph1):f2
50u UNBLKGRAD

p16:gp12*1.77             ;purging gradient between experiments
d16                       ;gpz12 = 17.13%

if "l8 %4 < 2"

{
20u

(p13 ph19):f1        ;p13 = (p1*l0/18) and l0 = 7, corresponds to v = 20 degree in BIG-BIRD
d26 pl0:f2
(p2 ph1)
(p31:sp18 ph1):f2     ;J-compensated adiabatic pulse during BIG-BIRD - H2L sweep!
d27 pl12:f2
(p1 ph20):f1

d22 cpd2:f2             ;with 13C-multiplicity editing
TAU do:f2
4u pl2:f2
(p3 ph3):f2

TAU9 pl0:f2         ;TAU9 = d2 - p29
(p29:sp20 ph1):f2   ;J-compensated adiabatic pulse - L2H sweep, 0.97ms!
d0
(p2 ph1)
d0
p16:gp1*EA1
TAU8
4u
(p29:sp20 ph1):f2   ;J-compensated adiabatic pulse - L2H sweep, 0.97ms!
4u pl2:f2
(p3 ph5):f2
p16:gp1*EA1*-1
TAU4 pl12:f2
d29 cpd2:f2

}
else
{

20u

(p13 ph19):f1          ;BIG-BIRD
d26 pl0:f2
(p2 ph1)
(p31:sp18 ph1):f2      ;J-compensated adiabatic pulse during BIG-BIRD - H2L sweep!
d27 pl12:f2
(p1 ph20):f1
```

```
d24 cpd2:f2            ;without 13C-multiplicity editing
TAU do:f2
4u pl2:f2
(p3 ph3):f2

d0
(p2 ph1)
d0
p16:gp1*EA1
d16 pl0:f2
(p29:sp20 ph1):f2      ;J-compensated adiabatic pulse
DELTA
p16:gp1*EA1*-1
d16
4u pl0:f2
(p29:sp20 ph1):f2     ;J-compensated adiabatic pulse
4u pl2:f2
(p3 ph5):f2
TAU pl12:f2
d25 cpd2:f2

}

4u do:f2
4u pl2:f2

(p1 ph2) (p3 ph1):f2

if "l9 %2 == 1"

{                         ;with 1B/2B-editing !!

p16:gp1*EA5
d16
DELTA5 pl0:f2
(p2 ph1):f1
(p30:sp19 ph1):f2     ;J-compensated adiabatic pulse during editing - H2L sweep, 1.92 ms
p16:gp1*0.75
d16
DELTA8 pl12:f2

}
else                      ;without 1B/2B-editing!
{

p16:gp1*EA5
d16
DELTA5 pl0:f2
(p2 ph1):f1
p16:gp1*0.75
d16
DELTA7 pl12:f2

}

4u cpd2:f2
4u BLKGRAD


go=2 ph31
d11 do:f2 wr #0 if #0 zd


  1m iu9
  1m iu8
  1m igrad EA2
  1m igrad EA3
```

```
  1m igrad EA4
  1m igrad EA5


lo to 3 times 2

 1m id10
 1m dd19
 1m ip24*2
 1m ip30*2
 1m ru9


 lo to 4 times 2

 1m igrad EA1
 1m ru8

 lo to 5 times 2

  1m id0
  1m dd22
  1m dd29
  1m dd24
  1m dd25
  1m ip3*2
  1m ip31*2

lo to 6 times l3              ;l3 = TD1/24


exit

ph1=0
ph14=0 2 2 0
ph15=0 0 2 2

ph2=1
ph3=0 0 2 2
ph5=0 2
ph6=0

ph22=0 0 0 0 2 2 2 2
ph23=0 0 2 2
ph24=0 2
ph25=0 0 0 0 0 0 0 0 2 2 2 2 2 2 2 2

ph16=(8) 1   ;BIG-BIRD phases - ph16-ph20
ph17=(8) 5

ph18=(72) 2

ph19 = ph16 - ph18
ph20 = ph17 + ph18

ph29=0 2
ph30=0 2 0 2 0 2 0 2 2 0 2 0 2 0 2 0
ph31=0 2 2 0


;pl1 : f1 channel - power level for pulse (default)
;pl2 : f2 channel - power level for pulse (default)
;pl12: f2 channel - power level for CPD/BB decoupling
;p1 : f1 channel - 90 degree high power pulse
;p2 : f1 channel - 180 degree high power pulse
;p3 : f2 channel - 90 degree high power pulse
;p16: homospoil/gradient pulse [1 msec]
```

```
;p24: f2 channel - 180 degree shaped pulse for refocussing
; = 2msec for Crp60comp.4
;d0 : incremented delay (2D) [3 usec]
;d10 : incremented delay (2D)   [3 usec]

;d11: delay for disk I/O [5-30 msec]
;d16: delay for homospoil/gradient recovery
;d6 : delay for evolution of long range couplings

;d19: decremented delay (2D)
;d22: decremented delay (2D)
;d21: constant time (evolution of J(HH)) [16 - 23 msec]
;d29: decremented delay (2D)
;d24: decremented delay (2D)
;d25: decremented delay (2D)

;cnst2: = 113
;cnst6: = 1J(XH)min
;cnst7: = 1J(XH)max
;cnst14: = J(XH) long range

;inf1: 1/SW(X) = 2 * DW(X)
;in0: 1/(2 * SW(X)) = DW(X)
;in10: 1/(2 * SW(X)) = DW(X)
;in19: = in0
;in22: = in0
;in29: = in0
;in24: = in0
;in25: = in0

;nd0: 2
;ns: 2 * n
;ds: >= 16
;td1: number of experiments
;FnMODE: UNDEFINED


;cpd2: decoupling according to sequence defined by cpdprg2
;pcpd2: f2 channel - 90 degree pulse for decoupling sequence

; ~~~~~~~~~~~~~~ WaveMaker Shapes ~~~~~~~~~~~~~~~~~~~~~~~~~~~
;USE: wvm -a  command in TopSpin command line to generate CA-WURST adiabatic shape pulses

;sp18:wvm:ad180Ccawurst: cawurst-20(240 ppm, 1.92 ms; H2L)

;sp20:wvm:ad180Ccawurstlh2: cawurst-20(240 ppm, 0.97 ms; L2H)

;sp19:wvm:ad180Ccawurst: cawurst-20(240 ppm, 1.92 ms; H2L)


;for z-only gradients:
;gpz1: 80%
;gpz2: -40.1% for C-13
;gpz3:  40.1%
;gpz4: 60%
;gpz5: -40%
;gpz6: -20%
;gpz11: 1
;gpz12 = 17.13% purging gradient
;gpz23 = 15%
;gpz14= -10%
;gpz15= -5%

;use gradient files:
;gpnam: SMSQ10.100
```

//

Pulse sequence code of NORD SEA XLOC(ZQ)– SEA XLOC(2Q)–2BOB(with 4-step phase editing) experiment for Bruker spectrometers (*Avance III and NEO systems*)

```
;nord_sea-xloc(zq)_sea-xloc(2q)_2bob(4)_ek_ows
;avance-version - tested on NEO system, TopSpin 4.0.2

;SEA XLOC
;2D H-1/X correlation via heteronuclear zero (ZQ) or double quantum (DQ/2Q) coherence
;recorded in two separate (SEA = separate echo-antiecho) experiments
;optimized for long range couplings
;with second order low-pass J-filter to suppress one-bond correlations
;no decoupling during acquisition
;using gradient pulses for coherence selection
;using adiabatic shaped pulses for inversion on f2 - channel

;T. Gyongyosi, T.M. Nagy, K.E. Kover & O.W. Sørensen, Chem. Commun. 54, 9781-9784 (2018)

;2BOB
;1B/2B heteronuclear correlation via heteronuclear zero and double quantum
;coherence with 13C multiplicity and 1B/2B updown time-shared 4-step phase cycle and
;gradient selection of Echo/Antiecho

;E. Kupce & O.W. Sørensen, Magn. Reson. Chem. 55, 515-518 (2017)
;N.T. Nyberg, J.Ø. Duus & O.W. Sørensen, J. Am. Chem. Soc. 127, 6154-6155 (2005)

;May 20, 2020 TMN, KEK

;This pulse sequence is part of
;Tamás Milán Nagy, Katalin E. Kövér, Ole W. Sørensen; NORD: NO Relaxation Delay NMR
;spectroscopy

;The pulse sequence has been coded for test purposes only and
;may contain errors.
;The functionality of the pulse sequence itself may differ depending on
;the hardware as well as the software used to execute it. Functionality
;on differing systems cannot be granted.
;Any use of this pulse sequence on a spectrometer is at your own risk.
;
;By using this pulse sequence, or any modification of it in any published material
;you agree to acknowledge the above-mentioned publication.

;NORD concatenation of THREE experiments: SEA XLOC(ZQ), SEA XLOC(2Q)
;and 2BOB with time-shared 4-step phase cycle of correlations
;according to 13C multiplicity and 1B vs. 2B correlation

;Double BANGO and BIG-BIRD elements include J-compensated adiabatic X-inversion pulse

;set FNmode - UNDEFINED and NBL=3


;PROCESSING:

;FIRST separate the three datasets using AU splitx
;Then separate the first and second(SEA XLOC ZQ and 2Q) data sets with AU split
;Co-add the resulting data sets and process each with xfb (set QF, mc in F1)
;The third data set corresponds to 2BOB with four-step phase cycle: 1B vs. 2B and also
;with carbon multiplicity editing, before processing use AU split (row 4!)
```

```
;Then the resulting four data sets can be processed with xfb (set echo-antiecho, pk)
;NOTE: F1 resolution in SEA XLOC is four times higher than that of 2BOB


;$CLASS=HighRes
;$DIM=2D
;$TYPE=
;$SUBTYPE=
;$COMMENT=

#include <Avance.incl>
#include <Grad.incl>
#include <Delayek.incl>

;use: wvm –a in TopSpin command line to setup adiabatic pulse parameters

"cnst2=113"


"cnst30=sfo1/(sfo2*4)"
"cnst31=(1-sfo2/sfo1)/(1+sfo2/sfo1)"

define list<gradient> EA1 = {cnst30 -cnst30}    ; 2BOB, phase sensitive
define list<gradient> EA5 = { 0.5 0.5 0.25 0.25}


"p2=p1*2"
"p4=p3*2"

"d0=3u"                        ;d0 for 2BOB
"d10=3u"                       ;d10 for SEA XLOC

"l9=0"
"l8=0"

"TAU=1s/(cnst6 + cnst7)"     ;2BOB


"d2=1s/(cnst2*2)"            ;for J-compensated adiabatic pulse - set CNST2 = 113

"TAU9 = d2 - p29"

"TAU1=d21/2-TAU-4u-d2-d0-p2/2"   ;d21: constant time for evolution of J(HH) (16-23 ms)
"d22=TAU1"

"TAU2=d21/2-TAU-p3-d0-p2/2-4u"   ;without 13C editing
"d24=TAU2"

"DELTA=d0*2+p2"
"TAU3=d21/2-p2/2-p3-TAU-d0-p16*2-d16*2-p29*2-DELTA-16u"   ;without 13C editing
"d25=TAU3"

"TAU8=d2-p16-p29-8u"
"TAU4=TAU-p16"
"d29=d21/2-d2-TAU-8u-d0-p2/2"  ;2BOB

"in0=inf1/2"
"in10=inf1/2"
"in19=in0"     ;SEA XLOC

"in22=in0"     ;2BOB
"in29=in0"     ;2BOB
"in24=in0"    ;2BOB
"in25=in0"    ;2BOB

"d6=1s/(cnst14*2)"     ;cnst14 – heteronuclear long-range coupling constant
```

```
"l3 = td1/24"    ;NBL = 3, data from three experiments are stored in one file

"DELTA1=1s/(2 * (cnst6 + 0.07 * (cnst7-cnst6) ) )"    ;2BOB
"DELTA3=1s/(2 * (cnst7 - 0.07 * (cnst7-cnst6) ) )"
"DELTA4 = d16 + p16"
"DELTA2=(DELTA1+DELTA3)/2-DELTA4"

"DELTA5=d2-p16-d16"
"DELTA8=d2-p16-d16-p30-8u"
"DELTA7=d2-p16-d16-8u"    ;without 1B/2B editing

"DELTA9=1s/(2 * (cnst6 + 0.146 * (cnst7-cnst6)) ) -p16-d16"  ;SEA XLOC
"DELTA12=1s/(2 * (cnst7 - 0.146 * (cnst7-cnst6)) ) -p16-d16"

"d23=1s/(cnst14*4)"
"d19=d23*2-p16*2-d16*2-d10"

"d26=0.5/cnst2"
"d27=d26-p31"

"p13=(p1*l0/18)"      ;l0 = 7, p13 = 35 degree, corresponds to v = 20 in BIG-BIRD
                      ;set ph18 accordingly

"p11 = (p1/90)*30"    ;p11 = 30 degree, corresponds to beta = 120 in second BANGO
"p12 = (p1/90)*20"    ;p12 = 20 degree, corresponds to beta = 140 in first BANGO

1 ze
2 d11 do:f2            ;d11=5-30 ms
  3m pl12:f2
3 5m
4 2m
5 7m do:f2
6 50u pl1:f1


  d11 do:f2 st0               ;NBL = 3, d11=30 ms NORD = NO Relaxation Delay

  d11 pl2:f2

  (p3 ph1):f2
  50u UNBLKGRAD
  p16:gp12*2.77
  d16                         ;purging gradient, gpz12 = 17.13%

  (p12 ph1):f1                ;SEA XLOC(ZQ) starts here with BANGO using 35 degree 1H pulse
                               ;resulting in -110 degree excitation of 1H-12C proton magn.
  d26 pl0:f2
  (p2 ph1)
  (p31:sp18 ph1):f2            ;J-compensated adiabatic pulse during BANGO - H2L sweep!
  d27
  (p12 ph1):f1

  DELTA9
  p16:gp4
  d16 pl2:f2
  (p3 ph1):f2
  DELTA12
  p16:gp5
  d16
  (p3 ph1):f2
  p16:gp6
  d16 pl2:f2
  d10
  (center (p2 ph1) (p4 ph1):f2 )
  d19
  p16:gp11                  ;gpz11 = 30 for ZQ-selection during t1
  d16
```

```
 (p3 ph14):f2
  p16:gp2
  d16 BLKGRAD
  d10
  d10
 (p3 ph15):f2

  goscnp ph29

 ;second experiment starts here: SEA XLOC(2Q)


12  d11 do:f2 st   ;d11=5-30 ms
13  d11 pl2:f2

   (p3 ph1):f2

  50u UNBLKGRAD
  p16:gp12*2.77               ;purging gradient between experiments
  d16                         ;gpz12 = 17.13%


   (p11 ph1):f1   ;SEA XLOC(2Q) starts here with BANGO using 30 degree 1H pulse resulting in
                  ;-120 degree excitation of 1H-12C proton magn.
  d26 pl0:f2
  (p2 ph1)
  (p31:sp18 ph1):f2   ;J-compensated adiabatic pulse during BANGO - H2L sweep!
  d27 pl2:f2
  (p11 ph1):f1

  DELTA9
  p16:gp4
  d16 pl2:f2
  (p3 ph1):f2
  DELTA12
  p16:gp5
  d16
 (p3 ph1):f2
  p16:gp6
  d16 pl2:f2
  d10
  (center (p2 ph1) (p4 ph1):f2 )
  d19
  p16:gp13                    ;gpz13 = 50 for 2Q selection
  d16
 (p3 ph14):f2
  p16:gp2
  d16 BLKGRAD
  d10
  d10
 (p3 ph15):f2

  goscnp ph29

;third experiment starts here - 2BOB with 4-step time-shared phase


22 d11 st         ;d11=5-30 ms
23 d11 do:f2      ;d11 only short delay between experiments
d11 pl2:f2

(p3 ph1):f2
50u UNBLKGRAD

p16:gp12*1.77             ;purging gradient between experiments
d16                       ;gpz12 = 17.13%
```

```
if "l8 %4 < 2"

{
20u

(p13 ph19):f1        ;p13 = (p1*l0/18) and l0 = 7, corresponds to v = 20 degree in BIG-BIRD
d26 pl0:f2
(p2 ph1)
(p31:sp18 ph1):f2     ;J-compensated adiabatic pulse during BIG-BIRD - H2L sweep!
d27 pl12:f2
(p1 ph20):f1

d22 cpd2:f2            ;with 13C-multiplicity editing
TAU do:f2
4u pl2:f2
(p3 ph3):f2

TAU9 pl0:f2         ;TAU9 = d2 - p29
(p29:sp20 ph1):f2   ;J-compensated adiabatic pulse - L2H sweep, 0.97ms!
d0
(p2 ph1)
d0
p16:gp1*EA1
TAU8
4u
(p29:sp20 ph1):f2   ;J-compensated adiabatic pulse - L2H sweep, 0.97ms!
4u pl2:f2
(p3 ph5):f2
p16:gp1*EA1*-1
TAU4 pl12:f2
d29 cpd2:f2

}
else
{

20u

(p13 ph19):f1          ;BIG-BIRD
d26 pl0:f2
(p2 ph1)
(p31:sp18 ph1):f2      ;J-compensated adiabatic pulse during BIG-BIRD - H2L sweep!
d27 pl12:f2
(p1 ph20):f1

d24 cpd2:f2            ;without 13C-multiplicity editing
TAU do:f2
4u pl2:f2
(p3 ph3):f2

d0
(p2 ph1)
d0
p16:gp1*EA1
d16 pl0:f2
(p29:sp20 ph1):f2      ;J-compensated adiabatic pulse
DELTA
p16:gp1*EA1*-1
d16
4u pl0:f2
(p29:sp20 ph1):f2     ;J-compensated adiabatic pulse
4u pl2:f2
(p3 ph5):f2
TAU pl12:f2
d25 cpd2:f2

}
```

```
4u do:f2
4u pl2:f2

(p1 ph2) (p3 ph1):f2

if "l9 %2 == 1"

{                          ;with 1B/2B-editing !!

p16:gp1*EA5
d16
DELTA5 pl0:f2
(p2 ph1):f1
(p30:sp19 ph1):f2     ;J-compensated adiabatic pulse during editing - H2L sweep, 1.92 ms
p16:gp1*0.75
d16
DELTA8 pl12:f2

}
else                      ;without 1B/2B-editing!
{

p16:gp1*EA5
d16
DELTA5 pl0:f2
(p2 ph1):f1
p16:gp1*0.75
d16
DELTA7 pl12:f2

}

4u cpd2:f2
4u BLKGRAD


go=2 ph31
d11 do:f2 wr #0 if #0 zd


  1m iu9
  1m iu8
  1m igrad EA5


lo to 3 times 2

 1m id10
 1m dd19
 1m ip24*2
 1m ip30*2
 1m ru9


 lo to 4 times 2

 1m igrad EA1
 1m ru8

 lo to 5 times 2

 1m id0
 1m dd22
 1m dd29
 1m dd24
 1m dd25
```

```
 1m ip3*2
 1m ip31*2

lo to 6 times l3              ;l3 = TD1/24


exit

ph1=0
ph14=0 2 2 0
ph15=0 0 2 2

ph2=1
ph3=0 0 2 2
ph5=0 2
ph6=0

ph22=0 0 0 0 2 2 2 2
ph23=0 0 2 2
ph24=0 2
ph25=0 0 0 0 0 0 0 0 2 2 2 2 2 2 2 2

ph16=(8) 1   ;BIG-BIRD phases - ph16-ph20
ph17=(8) 5

ph18=(72) 2

ph19 = ph16 - ph18
ph20 = ph17 + ph18

ph29=0 2
ph30=0 2 0 2 0 2 0 2 2 0 2 0 2 0 2 0
ph31=0 2 2 0


;pl1 : f1 channel - power level for pulse (default)
;pl2 : f2 channel - power level for pulse (default)
;pl12: f2 channel - power level for CPD/BB decoupling
;p1 : f1 channel - 90 degree high power pulse
;p2 : f1 channel - 180 degree high power pulse
;p3 : f2 channel - 90 degree high power pulse
;p16: homospoil/gradient pulse [1 msec]
;p24: f2 channel - 180 degree shaped pulse for refocussing
; = 2msec for Crp60comp.4
;d0 : incremented delay (2D) [3 usec]
;d10 : incremented delay (2D)   [3 usec]

;d11: delay for disk I/O [5-30 msec]
;d16: delay for homospoil/gradient recovery

;d19: decremented delay (2D)
;d22: decremented delay (2D)
;d21: constant time (evolution of J(HH)) [16 - 23 msec]
;d29: decremented delay (2D)
;d24: decremented delay (2D)
;d25: decremented delay (2D)

;cnst2: = 113
;cnst6: = 1J(XH)min
;cnst7: = 1J(XH)max
;cnst14: = J(XH) long range

;inf1: 1/SW(X) = 2 * DW(X)
;in0: 1/(2 * SW(X)) = DW(X)
;in10: 1/(2 * SW(X)) = DW(X)
;in19: = in0
```

```
;in22: = in0
;in29: = in0
;in24: = in0
;in25: = in0

;nd0: 2
;ns: 2 * n
;ds: >= 16
;td1: number of experiments
;FnMODE: UNDEFINED


;cpd2: decoupling according to sequence defined by cpdprg2
;pcpd2: f2 channel - 90 degree pulse for decoupling sequence

; ~~~~~~~~~~~~~~~ WaveMaker Shapes ~~~~~~~~~~~~~~~~~~~~~~~~~~~
;USE: wvm -a  command in TopSpin command line to generate CA-WURST adiabatic shape pulses

;sp18:wvm:ad180Ccawurst: cawurst-20(240 ppm, 1.92 ms; H2L)

;sp20:wvm:ad180Ccawurstlh2: cawurst-20(240 ppm, 0.97 ms; L2H)

;sp19:wvm:ad180Ccawurst: cawurst-20(240 ppm, 1.92 ms; H2L)


;for z-only gradients:
;gpz1: 80%
;gpz2: -40.1% for C-13
;gpz3:  40.1%
;gpz4: 60%
;gpz5: -40%
;gpz6: -20%
;gpz11: 30%
;gpz13: 50%
;gpz12 = 17.13% purging gradient

;use gradient files:
;gpnam: SMSQ10.100
```

Brainstorming on the concept of Ernst angle in complex NMR experiments

The Ernst angle described in Ref. [1] and in the book by Ernst, Bodenhausen and Wokaun (EBW) refers to excitation by a single pulse, so how does that apply in case of an extended pulse sequence containing several pulses and delays? It is by no means fully equivalent but a useful analogy is to notice that after an extended pulse sequence there is still transverse magnetization to be detected and possibly also some z magnetization, as is the case after a single pulse. There can, of course, also be other coherences and spin orders, but they are ignored in the crude analogy.

Most complex NMR pulse sequences saturate the spin systems and thus leave no z magnetization at the end, so in the crude analogy the optimum delay (relaxation + acquisition) is about 1.3T_1, as for excitation by a single $\pi/2$ pulse (Table 4.3.1 in EBW) that leaves no z magnetization.

Fig. 4.3.2 in EBW links the optimum single pulse excitation angle to the ratio of relaxation + acquisition delay to T_1. One could then in the NORD approach look at that figure for a zero relaxation delay and from the applied acquisition time read out what the corresponding optimum single-pulse excitation angle β_{opt} is, and use that as an estimate for how much z relative to transverse magnetization should be targeted at the end of a NORD module.

However, in the single-pulse experiment the longitudinal and transverse magnetizations are straightforwardly $M_{SS}cos(\beta_{opt})$ and $M_{SS}sin(\beta_{opt})$, respectively, with M_{SS} being the steady-state magnetization prior to the excitation pulse. In contrast, a longer pulse sequence evitably entails a loss of some of the steady-state magnetization built up before the first pulse of the sequence. Thus even for equal relaxation delays a single-pulse and a longer pulse sequence have different "effective" steady-state magnetizations and the down scaling from M_{SS} in the longer pulse sequence can well be different for the longitudinal and tranverse components.

For this and other reasons the crude model should only be taken as a qualitative picture. It must also be kept in mind that β_{opt} depends on T_1 that varies across a molecule, so there is a compromise to be made.

All BANGO and BIG-BIRD angles applied in the experiments of the current paper have been optimized empirically. Once such a parameter set has been determined it can routinely be used on molecules with similar T_1 relaxation times without optimization.

Finally, it follows from the single-pulse analogy that the closer the acquisition time is to 1.3T_1 the less is to be gained by the NORD approach. However, acquisition times in small-molecule NMR are typically well shorter than 1.3T_1. Should it ever happen that the acquisition time is larger than 1.3T_1 NORD is not applicable.
